# Complete, Selective, and Stepwise Carious Tissue Removal for Deep Carious Lesions in Vital Permanent Teeth: A Systematic Review and Meta-Analysis

**DOI:** 10.3390/dj14090610

**Published:** 2026-09-20

**Authors:** Emile Laville, Alexandra Pinto Rodrigues, Paulo Mascarenhas

**Affiliations:** 1 Egas Moniz School of Health & Science, Quinta da Granja, 2829-511 Caparica, Portugal; 2 Egas Moniz Center for Interdisciplinary Research (CiiEM), Egas Moniz School of Health & Science, Quinta da Granja, 2829-511 Caparica, Portugal

**Keywords:** deep carious lesions, complete carious tissue removal, selective carious tissue removal, stepwise carious tissue removal, intraoperative pulp exposure, pulpal failure, restorative failure

## Abstract

**Background/Objectives**: This systematic review compared complete (CCR), selective (SCR), and stepwise (SWR) carious tissue removal in vital permanent teeth with deep carious lesions. **Methods**: PubMed/MEDLINE, Embase, Scopus, the Cochrane Library, and B-on were searched through 20 January 2026. Randomized trials were assessed with RoB 2, grouped by trial family, and analysed by distinct outcomes. Direct random-effects models were fitted only with at least three informative independent families, using REML and modified Knapp–Hartung inference. The protocol was registered in PROSPERO (CRD420261321894). **Results**: Eleven reports from seven families were included. For SCR versus SWR, intraoperative exposure occurred in 0/295 versus 12/288 teeth (pooled RR 0.11, 95% CI 0.003–4.64; *p* = 0.127; three families), and late pulpal failure at 12–24 months occurred in 11/254 versus 22/243 teeth (pooled RR 0.50, 95% CI 0.01–21.53; *p* = 0.510; I-squared = 72.2%; three families). Both outcomes had very low-certainty evidence. Other direct contrasts were study-specific, and the exploratory network was too sparse for reliable inference. **Conclusions**: Evidence was insufficient to demonstrate a difference or establish superior long-term pulpal or restorative outcomes.

## 1. Introduction

Dental caries remains one of the most prevalent chronic conditions worldwide and carries substantial functional, social, and economic consequences [1,2,3,4]. Operative approaches have evolved from traditional complete-excavation concepts [5,6] to contemporary tissue-preserving care [7,8,9,10,11,12,13].

For this review, a deep carious lesion was operationally defined as a radiographic lesion extending at least into the middle third of dentin and described by the trial investigators as deep or pulp-proximal, in a vital permanent tooth without clinical or radiographic evidence of irreversible pulpal or apical disease. Current guidance distinguishes deep lesions with a radiographically visible dentin barrier from extremely deep lesions without such a barrier [14]. The historical trial thresholds (at least the middle third, at least one half, more than two thirds, or at least three quarters of dentin) were accepted as variants within the review question but were not assumed to be biologically equivalent.

The three strategies were consistently defined. CCR (also termed non-selective removal) removes carious tissue down to hard dentin at the pulpal wall. SCR leaves soft or firm dentin near the pulp and places the definitive restoration in one visit. SWR initially retains soft dentin, provisionally seals the cavity, and plans re-entry for further excavation and definitive restoration [14,15,16,17,18,19,20].

Intraoperative pulp exposure is a procedural event that alters the planned treatment pathway, but it is not synonymous with irreversible loss of vitality. Diagnosis-led vital pulp therapy may include direct pulp capping or partial/full pulpotomy in selected teeth, whereas root canal treatment remains appropriate when the pulpal diagnosis or other clinical findings require it [14,21,22,23,24]. Exposure and subsequent pulpal or restorative failure should therefore be assessed as distinct outcomes.

Previous reviews differed in eligible dentitions and study designs, outcome definitions, follow-up windows, rare-event methods, and how repeated reports from the same randomized cohort were handled [12,25,26,27,28,29,30]. This review compared CCR, SCR, and SWR in vital permanent teeth, grouped repeated reports by randomized trial family, and assessed intraoperative exposure, late pulpal failure, and restorative failure separately. Composite clinical–radiographic outcomes were recorded descriptively when reported but were excluded from outcome-specific syntheses when their components could not be separated.

Direct randomized evidence was the primary basis for inference. A secondary exploratory network analysis of late pulpal failure at 12–24 months is reported in the Supplementary Materials for transparency; it was not used for treatment ranking or clinical conclusions because the network was sparse and clinically imbalanced.

## 2. Materials and Methods

### 2.1. Protocol and Registration

The protocol for this systematic review and meta-analysis was submitted to PROSPERO on 4 March 2026 and registered on 11 March 2026 (CRD420261321894). Reporting followed PRISMA 2020 and PRISMA-NMA [31,32]. The registered analytical plan included a composite treatment-success outcome, a primary frequentist network meta-analysis using DerSimonian–Laird estimation, and P-score treatment ranking, with pairwise analyses when data were sufficient. The analytical framework used in this review treats procedural exposure, late pulpal failure, and restorative failure as distinct outcomes; prioritizes direct randomized evidence; groups repeated reports by trial family; restricts the network to an exploratory supplementary analysis of late failure; uses REML with modified Knapp–Hartung inference; does not perform treatment ranking or study-level meta-regression; and pools only contrasts supported by at least three informative independent families. Differences between the PROSPERO record and the methods used in this review, together with their rationale and timing/status, are reported in Supplementary Table S12.

### 2.2. Eligibility Criteria

The population comprised patients of any age with vital permanent teeth affected by deep carious lesions. Eligible randomized clinical trials directly compared at least two of CCR, SCR, and SWR and reported a pulpal, restorative, clinical, or radiographic outcome.

The review-specific definition of deep caries was a radiographic lesion extending at least into the middle third of the dentin, described by the trial investigators as deep or pulp-proximal, in a vital permanent tooth without irreversible pulpal or apical disease. Trial-specific depth thresholds and diagnostic tests were extracted as potential effect modifiers. They were not considered equivalent, and the small number of independent families precluded credible depth-stratified pooling or meta-regression.

Intraoperative pulp exposure was treated as a procedural outcome. Late pulpal failure comprised irreversible pulpitis, pulp necrosis, apical pathology, endodontic treatment, or extraction for pulpal reasons after completion of the assigned procedure. Restorative failure comprised restoration loss, fracture, replacement, or the original trial’s defined restorative endpoint. Composite clinical–radiographic outcomes were recorded descriptively and excluded from outcome-specific syntheses when their components could not be separated.

Post-treatment outcomes required at least 12 months of follow-up; intraoperative exposure was eligible irrespective of follow-up because it occurred during the assigned procedure. Publications in English, Portuguese, or French were eligible. Studies restricted to primary dentition, non-randomized designs, baseline irreversible pulpitis, pulp necrosis, relevant apical disease, or previous endodontic treatment were excluded.

Multiple publications from a single randomized cohort were grouped into a trial family. One eligible estimate per family, outcome, follow-up window, and direct contrast was included in any synthesis. Adjusted time-to-event estimates were retained in their original metric and were not pooled with binary risk ratios.

The registered population included permanent teeth in children, adolescents, and adults and did not explicitly specify a root-maturity restriction. As a post-registration scope clarification, reports explicitly confined to immature permanent teeth were excluded when root maturity was not reported by treatment arm and no compatible subgroup could be extracted separately; chronological age alone was not an exclusion criterion. The exact date of this clarification was not preserved and is disclosed in Supplementary Table S12.

### 2.3. Literature Search Strategy

PubMed/MEDLINE, Embase, Scopus, the Cochrane Library, and B-on were searched from inception to 20 January 2026. These sources provided complementary biomedical and dental indexing, multidisciplinary citation coverage, controlled-trial and review records, and access via the Portuguese B-on discovery service. Backward and forward citation tracking was conducted for the included reports.

Supplementary Methods S1.1 and S1.2 and Table S1 document the information sources, recorded search dates and yields, preserved search concepts, and retrospectively reconstructed database-specific search strings. No publication-year limit was applied. Human-study and randomized-trial filters were used when available, but exact historical, interface-specific filters cannot be verified. No dedicated searches of trial registries, gray-literature databases, thesis repositories, conference-proceeding databases, or other unpublished-study sources were conducted. The three-language restriction is acknowledged as a potential source of language bias.

### 2.4. Study Screening

All records were imported into Rayyan [33]. Candidate duplicates flagged by Rayyan were manually verified before removal [34]. Two reviewers independently screened titles and abstracts and assessed full texts; eligibility decisions were made by reviewers and not delegated to automated predictions [35]. Disagreements were resolved by discussion or, when necessary, by consulting a third reviewer. Reports not retrieved and excluded full texts were recorded separately (Supplementary Table S2).

### 2.5. Data Collection and Extraction

Data extraction was conducted independently by two reviewers using a standardized extraction form. The form was piloted on a subset of included studies before full data extraction. Discrepancies were resolved through discussion or, when necessary, by consulting a third reviewer.

The extracted data included report and trial-family identifiers, country, publication year, study design, participant and tooth counts, lesion-depth criteria, baseline pulpal status, intervention and comparator definitions, follow-up duration, attrition, outcome definitions, analysis population, arm-level events and denominators, and adjusted effect estimates, when reported. Outcomes were extracted as separate variables for intraoperative pulp exposure, late pulpal failure, restorative failure, and overall clinical–radiographic failure. Each outcome was linked to its exact denominator and analysis population. Exposures occurring during treatment were separated from subsequent pulpal events, and events excluded after randomization were not silently omitted from strategy-level analyses. When a publication reported an adjusted hazard ratio or another time-to-event estimate, that estimate was analyzed in its published metric; survival percentages were not converted into binary event counts. Double-zero comparisons were retained in event tables and risk-difference sensitivity analyses but did not contribute to risk-ratio pooling.

The treated tooth was the unit of analysis because most trials reported tooth-level data. Trial reports in which a single participant could contribute more than one tooth were flagged as clustered. When an appropriately adjusted estimate was available, it was preferred; otherwise, sampling variances were adjusted via a design-effect sensitivity analysis using a prespecified working intraclass correlation coefficient. Reports from the same trial family and repeated follow-up assessments were never treated as independent studies in the primary direct-evidence meta-analyses.

### 2.6. Risk-of-Bias Assessment

Risk of bias for each eligible report and outcome was assessed using the Cochrane Risk of Bias 2 (RoB 2) tool [36]. The assessment covered bias arising from the randomization process, deviations from intended interventions, missing outcome data, outcome measurement, and selection of the reported result.

Two reviewers independently assigned domain-level and overall judgments of low risk, some concerns, or high risk. Reports from the same randomized trial family were assessed separately if they reported distinct outcomes or follow-up results; risk-of-bias judgments were not automatically transferred across reports. Disagreements were resolved through discussion and, when necessary, by consulting a third reviewer.

### 2.7. Data Analysis

All analyses used R version 4.5.2 and a reproducible, script-based workflow. Primary syntheses relied on randomized evidence. Binary effects were reported as risk ratios (RRs) with 95% confidence intervals (CIs); a 0.5 continuity correction was applied only to comparisons with a zero cell. Double-zero comparisons were retained in event tables and risk-difference sensitivities but did not inform RR pooling. Adjusted time-to-event effects were retained as hazard ratios and were not combined with RRs. Individual-study and pooled estimates were tabulated in outcome-specific tables and displayed in forest plots on logarithmic scales where appropriate.

Repeated reports were grouped by trial family, and no family contributed more than one estimate to an outcome-window-contrast synthesis. Pooling required at least three informative, independent families. Random-effects models used restricted maximum likelihood and modified Knapp–Hartung inference; the safeguard prevented the Knapp–Hartung variance adjustment from producing a CI narrower than the corresponding Wald CI. Heterogeneity was summarized using tau-squared, I-squared, and QE. Contrasts with one or two informative families were reported on a study-by-study basis.

For reports in which participants could contribute more than one tooth and no cluster-adjusted estimate was available, we inflated the sampling variance using the design effect 1 + (m − 1)ICC, where m is the mean number of analyzed teeth per participant for the relevant report and outcome, and ICC is the intraclass correlation coefficient, representing the assumed correlation between tooth-level outcomes contributed by the same participant. No trial reported an empirical ICC; the primary analysis used an ICC of 0.10, with sensitivity analyses using ICC values of 0, 0.05, and 0.20 to represent independence, low clustering, and moderate clustering, respectively. These assumptions affected precision, not arm risks. Each family contributed one effect to the exploratory network, so its sampling covariance matrix was diagonal; a within-family correlation parameter (ρ) would be required only if correlated effects from the same family entered one model.

The secondary exploratory network was limited to RR-informative 12–24-month late pulpal failure and is reported in the Supplementary Materials. Transitivity was assessed qualitatively, and consistency and unrelated mean-effects models were compared using low-power Wald and likelihood-ratio tests. No ranking or meta-regression was performed. Full rare-event, clustering, network, inconsistency, and sensitivity methods are provided in Supplementary Methods S1.4–S1.8 and Tables S8–S15.

### 2.8. Certainty of Evidence

Direct-evidence certainty was assessed using GRADE [37]. Randomized evidence was downgraded when RoB 2 concerns were judged serious, including incomplete allocation reporting, operator awareness for excavation-dependent outcomes, missing or conditional outcome data, clustering not addressed in the original reports, and unavailable prospective analysis plans. The two pooled SCR-versus-SWR outcomes were rated as very low certainty due to serious risk of bias and very serious imprecision; late pulpal failure was also downgraded for inconsistency (Supplementary Table S13).

Confidence in the exploratory network was conservatively assessed using the CINeMA framework [38] across the domains of within-study bias, across-study bias, indirectness, imprecision, heterogeneity, and incoherence. All network comparisons were judged to have very low confidence and were therefore excluded from the primary conclusions; methods and domain judgments are reported only in the Supplementary Materials.

## 3. Results

### 3.1. Study Selection

The searches identified 1030 records. After manual verification and removal of 538 duplicates, 492 records were screened, 28 reports were sought, and 24 full texts were assessed; four reports could not be retrieved.

**Figure 1:**
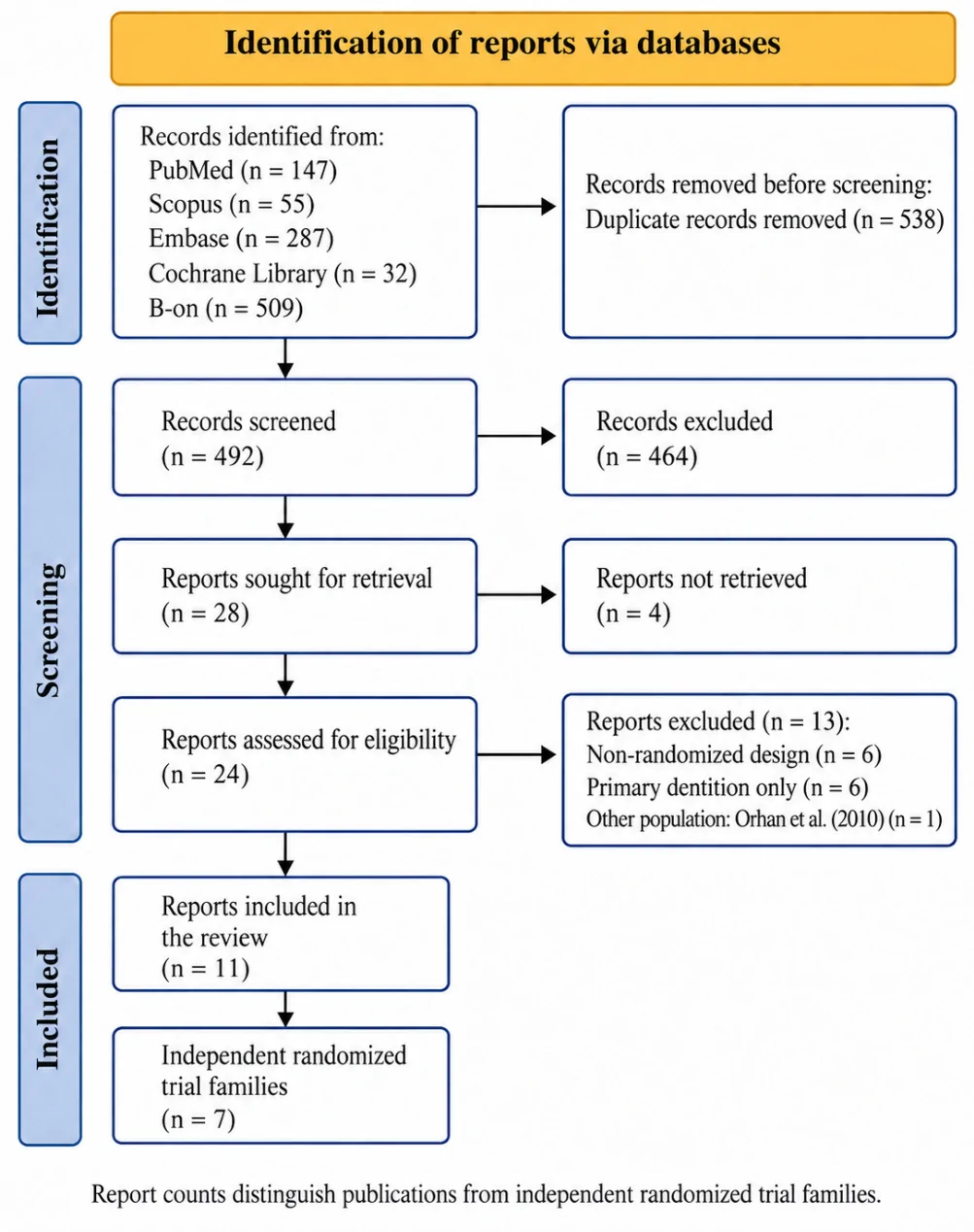
PRISMA 2020 flow diagram of record identification, report selection, and inclusion of randomized trial families. Orhan et al. (2010) [39].

Thirteen full-text reports were excluded: six had non-randomized designs, six were restricted to primary dentition, and Orhan et al. (2010) [39] was excluded separately. That report included primary molars and young permanent first molars; in the potentially eligible permanent-tooth subset, root maturity was not reported by treatment arm, and no subgroup compatible with the post-registration scope clarification was separately extractable. Eleven eligible reports represented seven independent randomized trial families. Repeated reports from the Bjørndal and Maltz families were retained for distinct outcomes or follow-up assessments but were not treated as independent trials (Figure 1; Supplementary Table S2).

### 3.2. Characteristics of Included Studies

The seven randomized trial families spanned Europe, Africa, South America, and Asia and included children, adolescents, and adults, primarily with permanent molars and premolars. Table 1 groups the 11 reports by family and summarizes clinically relevant characteristics of the population, lesion, diagnosis, excavation, restoration, and exposure management.

Historical lesion-depth thresholds ranged from at least the middle third to at least three-quarters of the dentin. Proximity to the pulp and the presence or absence of a radiopaque dentin barrier can influence exposure risk and prognosis; these thresholds were therefore treated as heterogeneous variants and potential effect modifiers, not as equivalent lesions. The family count was too small for reliable depth-stratified analysis.

Pulp tests, symptom exclusions, excavation endpoints, lining and restorative materials, management after exposure, analysis populations, and follow-up also varied. These differences informed GRADE, the supplementary transitivity assessment, and the decision to prioritize direct outcome-specific evidence.

### 3.3. Risk-of-Bias Assessment

**Figure 2:**
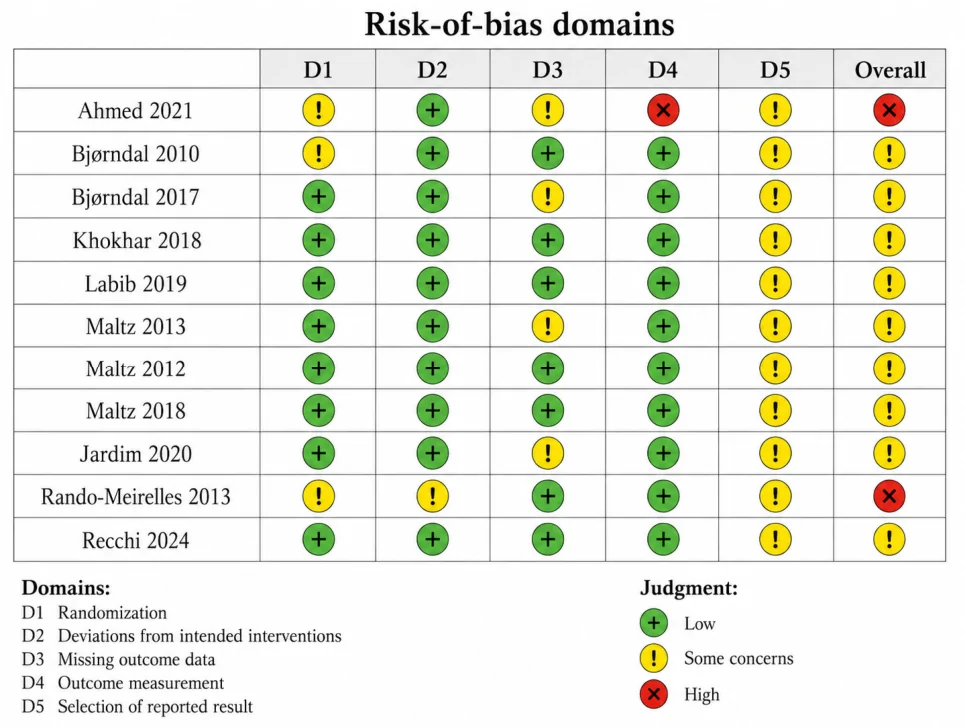
Report-level overview of risk-of-bias judgments for the 11 eligible reports [18,19,40,41,42,43,44,45,46,47,48] using the Cochrane RoB 2 tool. Outcome-specific departures that affected synthesis are described in the text.

RoB 2 judgments for the 11 eligible reports are summarized at the report level in Figure 2; when judgments differed by outcome, the outcome-specific judgment used in the synthesis took precedence. Ahmed 2021 [40] and Rando-Meirelles 2013 [47] were at high overall risk in the overview; the other nine reports raised some concerns, commonly because protocols or prespecified analysis plans were unavailable. The lack of long-term data affected the Bjørndal [19,41], Maltz [18,44,45], and Jardim [46] reports. Khokhar’s procedural overview [42] raised some concerns, whereas the late outcome was considered high risk because all 13 exposed CCR teeth were removed after randomization. These concerns led to a serious GRADE risk-of-bias downgrade for the primary pooled outcomes.

### 3.4. Structure of the Outcome-Specific Evidence

Only two contrasts met the requirement of at least three informative independent families for pooling: SCR versus SWR for intraoperative pulp exposure and SCR versus SWR for late pulpal failure at 12–24 months. All SCR-versus-CCR, SWR-versus-CCR, and restorative-failure estimates were reported study by study. Rando-Meirelles 2013 [47], Labib 2019 [43], and Maltz 2013 [44] contributed double-zero comparisons that were retained descriptively but did not inform RR pooling.

The two pooled comparisons and all primary study-specific binary direct estimates are summarized in Table 2 using the primary working ICC of 0.10. Adjusted time-to-event estimates were kept separate from binary RRs, and repeated long-term reports from the same trial family were not treated as independent confirmation.

### 3.5. Primary Pooled Direct Evidence

For intraoperative pulp exposure, three independent SCR-versus-SWR families reported 0/295 events with SCR and 12/288 with SWR. The pooled RR was 0.11 (95% CI 0.003–4.64; *p* = 0.127), with tau-squared = 0, I-squared = 0%, and QE = 0.046 (*p* = 0.977). Although the point estimate favored SCR, the modified Knapp–Hartung interval was compatible with both a large procedural benefit and harm; therefore, the evidence was insufficient to demonstrate a difference.

**Figure 3:**
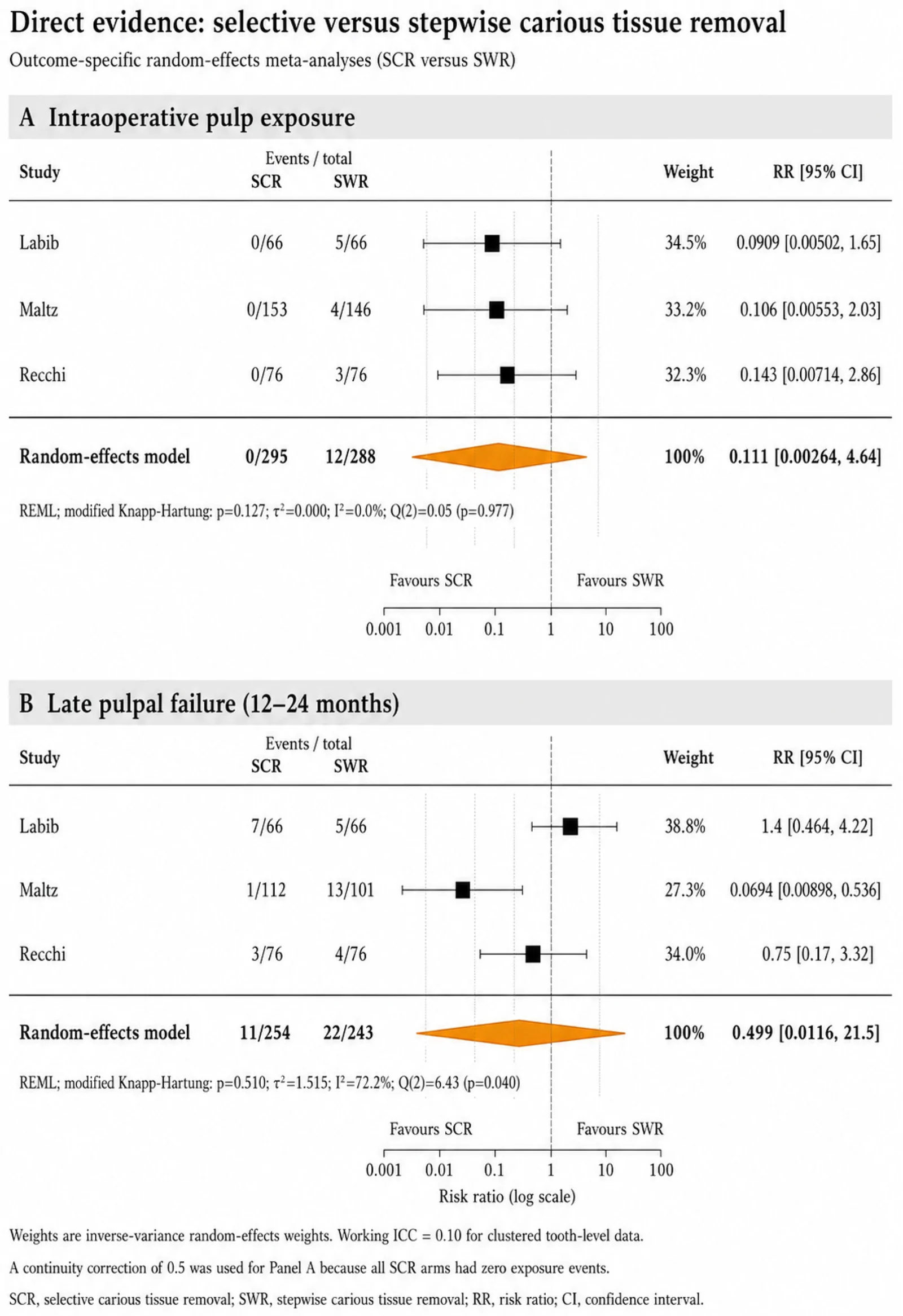
Direct random-effects meta-analyses comparing selective carious tissue removal (SCR) with stepwise removal (SWR). Panel (**A**) shows intraoperative pulp exposure, and Panel (**B**) shows late pulpal failure at 12–24 months [43,44,48]. Squares represent study-specific RRs, horizontal lines represent 95% CIs, and orange diamonds represent pooled RRs with 95% CIs. Values below 1 favor SCR. Models used REML with modified Knapp–Hartung inference and a working ICC of 0.10. A continuity correction of 0.5 was applied in Panel A because all SCR arms had zero exposure events.

For late pulpal failure at 12–24 months, three independent SCR-versus-SWR families reported 11/254 and 22/243 events, respectively. The pooled RR was 0.50 (95% CI 0.01–21.53; *p* = 0.510), with tau-squared = 1.52, I-squared = 72.2%, and QE = 6.43 (*p* = 0.040). The extreme imprecision and substantial heterogeneity precluded a reliable comparative conclusion (Figure 3).

### 3.6. Study-Specific Direct Evidence

For intraoperative pulp exposure, SCR versus CCR was not pooled. Ahmed 2021 [40] reported an RR of 0.07 (95% CI 0.004–1.12), and Khokhar 2018 [42] reported an RR of 0.04 (95% CI 0.002–0.63). SWR versus CCR was informed by the Bjørndal family [19,41] alone (RR 0.61, 95% CI 0.39–0.94). These are individual trial-family estimates and do not establish a pooled comparison-level effect.

For late pulpal failure, Ahmed 2021 [40] reported an RR of 5.00 (95% CI 0.25–99.95) for SCR versus CCR, whereas Rando-Meirelles 2013 [47] reported no events in either arm (RD 0.00, 95% CI −0.32 to 0.32). The Khokhar estimate, after all 13 exposed CCR teeth had been removed from follow-up, was retained only as conditional sensitivity evidence (RR 4.18, 95% CI 0.50–34.73). For SWR versus CCR, the single Bjørndal estimate was RR 0.96 (95% CI 0.45–2.04).

### 3.7. Restorative Failure and Adjusted Time-to-Event Estimates

For restorative failure at 12–24 months, Labib 2019 [43] and Maltz 2013 [44] reported no events in either arm, whereas Recchi 2024 [48] reported 4/72 versus 2/63 failures for SCR versus SWR (RR 1.75, 95% CI 0.32–9.49). No pooled RR was calculated. The double-zero RDs were 0.00 (95% CI −0.056 to 0.056) for Labib and 0.00 (95% CI −0.038 to 0.034) for Maltz. Current evidence was insufficient to support a comparative restorative conclusion.

Adjusted time-to-event estimates were reported separately and were not pooled across outcomes, follow-up windows, or repeated reports from the same trial family. Bjørndal 2017 [41] reported an HR of 0.67 (95% CI 0.45–1.05) for late pulpal failure with SWR versus CCR. The Maltz family reported HRs of 0.21 (95% CI 0.07–0.63) at 36 months and 0.38 (95% CI 0.23–0.63) at 60 months for SCR versus SWR. Jardim 2020 [46] reported an HR of 0.83 (95% CI 0.41–1.69) for restorative failure at 60 months.

Recchi et al. [48] also provided a secondary sensitivity estimate for restorative failure (HR 3.85, 95% CI 0.58–25.00) that did not explicitly account for patient-level clustering. The Labib composite HR was reported descriptively but excluded from the outcome-specific synthesis because it did not correspond to a single endpoint; no inseparable composite outcome qualified for outcome-specific synthesis. The long-term Bjørndal, Maltz, and Jardim estimates were also vulnerable to substantial attrition, and the two Maltz HRs arose from a single randomized family rather than from independent replication. Estimates are summarized in Table 3 and Supplementary Figure S1.

### 3.8. Sensitivity Analyses and Exploratory Network

ICC values from 0 to 0.20 did not alter the interpretation of the direct analyses. For the all-zero SCR exposure pattern, the no-correction RD was −0.036 (95% CI −0.083 to 0.012; *p* = 0.084). An exact conditional common-effect OR was 0.00 (95% CI 0.00–0.34; *p* < 0.001), but this method did not adjust for clustering and was not the primary analysis. Discordance in statistical significance across rare-event methods reinforced the need for cautious interpretation (Supplementary Tables S8 and S9).

The exploratory late-failure network is reported only in the Supplementary Materials. It contained very few family-level effects, showed marked clinical imbalance across direct comparisons, and yielded low-power inconsistency tests; all comparisons had very low CINeMA confidence. It did not support treatment ranking or alter the direct-evidence conclusions (Supplementary Tables S10–S15 and Figure S2).

### 3.9. Certainty of Evidence

GRADE rated both pooled SCR-versus-SWR outcomes as very low certainty (Table 4). For intraoperative exposure, the final modified Knapp–Hartung interval required a very serious imprecision downgrade in addition to the existing risk-of-bias downgrade. Late pulpal failure was further limited by substantial inconsistency. Other direct contrasts remained at low or very low certainty and were reported without pooling when fewer than three informative families were available.

The exploratory network remained very low confidence under CINeMA due to sparse evidence, clinical imbalance across contrasts, extreme imprecision, heterogeneity, and low-power incoherence assessment. These judgments did not change the primary reliance on direct evidence.

## 4. Discussion

This systematic review included 11 reports from seven randomized trial families. Only the SCR versus SWR comparisons for intraoperative exposure and for 12–24-month late pulpal failure met the requirement of three informative families for pooling; both estimates were very imprecise and rated as very low certainty. Other contrasts remained study-specific, and the supplementary exploratory network was not used for ranking.

### 4.1. Principal Findings and Interpretation

Separating intraoperative exposure from subsequent failure clarified that they address different clinical questions. SCR intentionally avoids further pulpal excavation, whereas SWR includes planned re-entry and additional excavation; therefore, a lower exposure point estimate represents, at most, a possible procedural advantage [15,16,19,43,44]. With only three families and an interval compatible with substantial benefit and harm, the evidence was insufficient to demonstrate a difference and did not establish overall clinical superiority.

Late pulpal and restorative outcomes remained highly uncertain. In Bjørndal 2017 [41], 239/314 participants were assessed at five years, and 220/314 were included in the principal Cox dataset. In Maltz 2018 [18], 229/299 randomized teeth contributed at least one observation to survival modeling, but only 121/299 were examined exactly at five years; the related Jardim restorative analysis included 172/299 restorations. Although censoring and frailty models use all available follow-up information, they cannot fully correct for bias when loss to follow-up is related to prognosis or differs between treatment groups. Likewise, repeated reports from the Maltz study family should not be interpreted as independent confirmation, because they may draw on overlapping participants or follow-up periods.

### 4.2. Comparison with Existing Literature

The findings align with the biological basis of conservative carious tissue management. Arrest of a sealed lesion is associated with reduced nutrient availability and decreased bacterial activity, indicating that durable isolation from the oral environment is central to disease control [17,49,50,51,52,53]. The present outcome-specific results suggest that excavation strategy may have its most immediate effect on the risk of procedural pulp exposure, whereas subsequent pulpal and restorative outcomes are also influenced by baseline pulpal condition, restoration quality, patient attendance, and the success of the definitive seal.

Previous reviews reached differing conclusions because they differed in eligible dentitions and designs, family grouping, composite versus outcome-specific definitions, follow-up windows, zero-event handling, and network methods [28,29,30]. This review grouped repeated reports by randomized family; separated procedural exposure, late pulpal failure, and restorative failure; retained one estimate per family/outcome/window/contrast; kept double-zero comparisons descriptive or as risk differences; retained adjusted HRs in their published metric; and used modified Knapp–Hartung inference only when at least three informative families were available.

### 4.3. Clinical Implications

Avoiding exposure should not be equated with preserving long-term vitality or tooth survival. Contemporary management is diagnosis-led: depending on symptoms, pulpal status, hemostasis, tissue appearance, restorability, and patient factors, a carious exposure may be managed with direct capping, partial or full pulpotomy, or root canal treatment [14,23,24]. Evolving vital-pulp therapy also limits the use of exposure as a surrogate for inevitable loss of vitality.

SCR may be considered for a vital permanent tooth with a favorable pulpal diagnosis when the lesion is pulp-proximal, a sound peripheral seal and a durable definitive restoration are achievable, and one-visit completion is advantageous [14,20]. This is a contextual clinical consideration, not a subgroup effect demonstrated by this review.

SWR may remain an option when staged reassessment or planned re-entry is clinically justified and reliable attendance at the second visit can be expected. CCR may retain a limited role when a lesion is less pulp-proximal, the anticipated biological cost is low, and removal to hard dentin is needed for a sound restorative procedure. Extremely deep lesions require planning for possible vital-pulp therapy rather than treating exposure as an automatic failure [14,20,23,24].

The evidence does not support a universal ranking of CCR, SCR, and SWR, a claim of equivalence or non-inferiority, or a prescriptive preference based solely on the exposure outcome. Decisions should integrate pulpal diagnosis, lesion depth, tooth restorability, seal quality, operator expertise, patient preferences, and the likelihood of completing planned follow-up.

### 4.4. Strengths and Limitations

The principal strength was the outcome-specific extraction and auditable analysis of randomized evidence. Reports were linked to trial families; pooling required at least three informative, independent families; adjusted HRs were kept separate from RRs; double-zero and rare-event choices were made explicit; and the influence of plausible within-participant clustering was examined. The reproducible computational workflow includes input and script hashes, outputs, figures, and session information.

The evidence base was small and uneven. Seven independent families provided insufficient power for stable small-sample inference, consistency testing, or exploration of effect modifiers such as lesion depth, age, tooth type, restorative material, and exposure management. Clinical and methodological heterogeneity spanned depth thresholds, pulpal diagnostic criteria, excavation endpoints, lining/restorative protocols, exposure management, outcome definitions, and analysis denominators. Khokhar excluded all 13 exposed CCR teeth from late follow-up, conditioning on a post-randomization event related to treatment and prognosis, potentially inflating observed CCR success. Long-term attrition and repeated reports further reduced the independence and applicability of the evidence.

Review-level limitations included registration after completion of the searches and screening; a root-maturity eligibility clarification not specified in the PROSPERO record; restriction to English, Portuguese, and French; no dedicated searches of trial registries, gray-literature databases, thesis repositories, conference-proceeding databases, or other unpublished-study sources; four reports not retrieved; and inability to assess small-study effects. Native platform search histories were not retained; consequently, the strings in Supplementary Methods S1.2 are retrospective reconstructions based on preserved concepts and applicable syntax, and exact historical, interface-specific filters cannot be verified. The date and yield of citation tracking were also not separately preserved. Additional limitations included differences between the registered and implemented analytical methods, working ICC assumptions, possible publication bias, low-power network diagnostics, and changes in vital-pulp therapy across the study period.

### 4.5. Implications for Future Research

Future randomized trials should prespecify and report intraoperative pulp exposure, late pulpal failure, restorative failure, and tooth survival as distinct outcomes. Arm-level denominators should remain traceable to randomization, and reasons for exclusions and losses should be reported by treatment group. Studies should report both participant and tooth counts, account for clustering when participants contribute multiple teeth, and use standardized baseline pulpal diagnoses and lesion-depth criteria. Harmonized assessments at 12, 24, and 60 months would improve comparability.

Adequately powered head-to-head or three-arm trials should also standardize excavation endpoints, lining and restorative protocols, and management after accidental exposure. Adherence to the planned second visit is especially important for SWR and should be reported as part of the treatment strategy. Prospective registration, accessible statistical analysis plans, patient-centered outcomes, cost-effectiveness measures, and sharing de-identified data and analysis code would strengthen future evidence and enable more reliable investigation of treatment-effect modifiers.

## 5. Conclusions

For SCR versus SWR, the point estimate suggested fewer intraoperative exposures with SCR, but the very low-certainty evidence and an extremely wide modified Knapp–Hartung confidence interval were insufficient to demonstrate a difference.

Evidence was also insufficient to determine whether any procedural advantage translates into better long-term pulpal or restorative outcomes. Adjusted long-term HRs were derived from individual trial families and were not pooled; the exploratory network was too sparse and clinically imbalanced to support reliable inference or ranking. The available evidence does not establish equivalence, non-inferiority, or a universal treatment hierarchy. Further adequately powered randomized trials with standardized outcome definitions, explicit management of exposure, clustering-adjusted analyses, and harmonized follow-up are needed before firm clinical recommendations can be made.

## Figures and Tables

**Table 1:** Clinical characteristics of the seven randomized trial families represented by 11 eligible reports.

Trial Family/Reports	Population and Tooth Type	Lesion and Baseline Pulp Criteria	SCR Endpoint	SWR Endpoint	CCR Endpoint	Liner/Restoration and Exposure Management	Follow-Up/Analysis Notes
Ahmed 2021 [40]	Pakistan; permanent molars; CCR 23.4 ± 5.5 y, SCR 25.6 ± 4.9 y; 60 analysed (70 reportedly enrolled; disposition of 10 NR)	≥1/2 dentine; radiograph, cold and electric testing; detailed symptom thresholds NR	Wet dentine retained near pulp	Not studied	Detector dye until unstained	RMGIC + etched incremental composite; all reported exposures pulpotomized	18 mo; analysed groups 30 + 30
Bjørndal family: 2010/2017 [19,41]	Denmark/Sweden; adults; one permanent tooth/person; anterior teeth, premolars and molars; median 29 y	≥75% dentine with radiodense zone; cold/EPT response; no apical lesion or prolonged/night pain	Not studied	Soft dentine turned to hard at 8–12 wk	Hard dentine at first visit	Dycal/Ketac Molar → OptiBond Solo Plus/Herculite XRV; nested Dycal cap vs. partial pulpotomy	12 mo: 292 analysed (143 SWR, 149 CCR); 22 lost (13/9). At 5 y: 239 assessed; Cox n = 220.
Khokhar 2018 [42]	India; mature mandibular permanent first/second molars; 14–54 y; 136 after initial loss	≥1/2 dentine; cold + EPT vital; no apical lesion or irreversible symptoms	Soft wet dentine retained	Not studied	Repeated detector dye until unstained	Fuji Lining LC + Tetric N-Ceram; 13 CCR exposures received capping/pulpotomy or root-canal treatment and were excluded from late success	18 mo; exposure analysis retained; late estimate conditional/high RoB
Labib 2019 [43]	Egypt; permanent premolars/molars; 18–47 y; 132 teeth	>2/3 dentine with radiopaque layer; Endo-Ice response; no prolonged pain, percussion pain, or apical lesion	Soft dentine at first visit; no further removal	Soft turned to firm at 3–4 mo	Not studied	Fuji IX/Equia → Single Bond Universal/Filtek Z350 XT; NaOCl haemostasis + Dycal direct cap	12 mo interim analysis
Maltz family: 2012/2013/2018; Jardim 2020 [18,44,45,46]	Brazil; permanent molars; 6–53 y; 299 teeth/233 participants; first molars 62%, second 33%, third 5%	≥1/2 dentine; positive cold; no spontaneous pain, percussion pain, or apical lesion	Disorganized soft dentine retained; one-visit seal	Same first stage; remaining caries removed at ≈90 d	Not studied	Vitro Fil + amalgam/composite; SWR Dycal + IRM then definitive restoration; 4 SWR exposures: 3 caps, 1 root-canal treatment	18 mo available-case: 112 SCR + 101 SWR; 86/299 not analysed. At 5 y: 121 examined; 229 contributed censored data.
Rando-Meirelles 2013 [47]	Brazil; permanent first/second molars; 12–17 y; 18 teeth	≥middle third dentine; cold response; no spontaneous/exaggerated pain or apical lesion	Pulpal caries retained	Not studied	Hard dentine	SCR Vidrion F; CCR Dycal + Vidrion F; both Single Bond/Filtek Z350; exposure protocol NR	24 mo: 16 analysed (8 + 8); 2 losses, reasons NR. No exposure reported.
Recchi 2024 [48]	Brazil; posterior permanent teeth; 9–55 y; 152 randomized	≥50% dentine; positive Endo-Ice; no spontaneous pain, percussion pain, or apical lesion	Soft dentine retained	Soft turned to firm at ≈90 d	Not studied	Liner randomization (Dycal vs. adhesive); Single Bond Universal/Filtek Z350; 3 SWR exposures direct-capped	18 mo restorative: 72 SCR + 63 SWR; 17/152 not analysed; arm-specific disposition incomplete.

Note: Bjørndal (2010 and 2017) [19,41] are reports from a single-trial family. Maltz 2012, Maltz 2013, Maltz 2018, and Jardim 2020 [18,44,45,46] are reports from the multicenter NCT00887952 trial family and were not treated as independent trials. Abbreviations used in Table 1: EPT, electric pulp testing; IRM, intermediate restorative material; NaOCl, sodium hypochlorite; NR, not reported; RMGIC, resin-modified glass-ionomer cement; d, days; mo, months; wk, weeks; y, years.

**Table 2:** Outcome-specific direct evidence under the primary working ICC of 0.10.

Outcome/Contrast	Evidence	Events (First vs. Second)	RR (95% CI)	Analysis and Interpretation
Intraoperative pulp exposure: SCR vs. SWR	3 families [43,44,48]	0/295 vs. 12/288	0.11 (0.003–4.64)	Pooled REML + modified Knapp–Hartung; *p* = 0.127; tau-squared = 0; I-squared = 0%. Evidence is insufficient to demonstrate a difference.
Late pulpal failure, 12–24 months: SCR vs. SWR	3 families [43,44,48]	11/254 vs. 22/243	0.50 (0.01–21.53)	Pooled REML + modified Knapp–Hartung; *p* = 0.510; tau-squared = 1.52; I-squared = 72.2%. Evidence is insufficient to demonstrate a difference.
Intraoperative pulp exposure: SCR vs. CCR	2 families [40,42]	Ahmed: 0/30 vs. 7/30; Khokhar: 0/67 vs. 13/69	Ahmed: 0.07 (0.004–1.12); Khokhar: 0.04 (0.002–0.63)	Study-specific estimates only; no pooling (<3 informative families).
Intraoperative pulp exposure: SWR vs. CCR	1 family [19]	25/143 vs. 43/149	0.61 (0.39–0.94)	Single-family estimate; not pooled.
Late pulpal failure, 12–24 months: SCR vs. CCR	2 families [40,47]; 1 RR-informative	Ahmed: 2/30 vs. 0/30; Rando-Meirelles: 0/8 vs. 0/8	Ahmed: 5.00 (0.25–99.95); Rando-Meirelles: RR not estimable	Study-specific evidence only; no pooling.
Late pulpal failure, 12–24 months: SWR vs. CCR	1 family [19]	12/143 vs. 13/149	0.96 (0.45–2.04)	Single-family estimate; not pooled.
Restorative failure, 12–24 months: SCR vs. SWR	3 families [43,44,48]; 1 RR-informative	Recchi: 4/72 vs. 2/63; Labib: 0/66 vs. 0/66; Maltz: 0/112 vs. 0/101	Recchi: 1.75 (0.32–9.49); other RRs not estimable	Study-specific evidence only; no pooling.

Note: RR values below 1 favor the first-listed treatment. Pooled effects were calculated only for contrasts with at least three informative, independent families. Double-zero comparisons were retained for descriptive purposes and as RD sensitivity analyses.

**Table 3:** Adjusted hazard ratios retained in the reported time-to-event metric.

Report	Outcome	Contrast	Months	HR (95% CI)	Analytical Status
Bjørndal—5-year report (2017) [41]	Late pulpal failure	SWR vs. CCR	60	0.67 (0.45–1.05)	Study-specific direct; descriptive (not pooled)
Maltz—3-year report (2012) [45]	Late pulpal failure	SCR vs. SWR	36	0.21 (0.07–0.63)	Study-specific direct; descriptive (not pooled)
Maltz—5-year report (2018) [18]	Late pulpal failure	SCR vs. SWR	60	0.38 (0.23–0.63)	Study-specific direct; descriptive (not pooled)
Jardim—5-year restorative report (2020) [46]	Restorative failure	SCR vs. SWR	60	0.83 (0.41–1.69)	Study-specific direct; descriptive (not pooled)
Recchi—18-month report (2024) [48]	Restorative failure	SCR vs. SWR	18	3.85 (0.58–25.00)	Secondary sensitivity; descriptive (not pooled)

Note: Maltz 2012 [45], Maltz 2018 [18], and Jardim 2020 [46] are part of the same multicenter trial family. Their estimates represent different follow-up reports or outcomes and were not treated as independent observations in a pooled model. Outcome-specific GRADE judgments for these adjusted estimates are provided in Supplementary Table S14.

**Table 4:** Summary of GRADE certainty for procedural and 12–24-month binary direct evidence.

Outcome/Contrast	Effect (95% CI)	Outcome-Specific Analysed Teeth	Certainty	Main Reasons
Intraoperative exposure: SCR vs. SWR	Pooled RR 0.11 (0.003–4.64)	583 teeth; 3 families [43,44,48]	Very low	Serious risk of bias; very serious imprecision
Late pulpal failure, 12–24 months: SCR vs. SWR	Pooled RR 0.50 (0.01–21.53)	497 teeth; 3 families [43,44,48]	Very low	Serious risk of bias; serious inconsistency; very serious imprecision
Intraoperative exposure: SCR vs. CCR	Ahmed RR 0.07 (0.004–1.12); Khokhar RR 0.04 (0.002–0.63)	196 teeth; 2 families [40,42]; not pooled	Very low	Serious risk of bias; very serious imprecision
Intraoperative exposure: SWR vs. CCR	RR 0.61 (0.39–0.94)	292 teeth; 1 family [19]	Low	Serious risk of bias; serious imprecision
Late pulpal failure, 12–24 months: SCR vs. CCR	Ahmed RR 5.00 (0.25–99.95); one double-zero family	76 teeth; 2 families [40,47]; not pooled	Very low	Serious risk of bias; very serious imprecision
Late pulpal failure, 12–24 months: SWR vs. CCR	RR 0.96 (0.45–2.04)	292 teeth; 1 family [19]	Very low	Serious risk of bias; very serious imprecision
Restorative failure, 12–24 months: SCR vs. SWR	Recchi RR 1.75 (0.32–9.49); two double-zero families	480 teeth; 3 families [43,44,48]; not pooled	Very low	Serious risk of bias; very serious imprecision

Note: Risk-of-bias judgments were based on the outcome-specific RoB 2 assessments. Certainty ratings incorporated the modified Knapp–Hartung confidence intervals and required at least three informative, independent families for pooling. Detailed domain judgments are provided in Supplementary Table S13.

## Data Availability

The outcome-specific dataset, analysis R script, complete checked output directory, primary forest figure, excluded-studies file, checksums, run provenance, and session information are available in Zenodo [54].

## Supplementary Materials

The following supporting information can be downloaded at: https://www.mdpi.com/article/10.3390/dj14090610/s1. Supplementary Methods S1.1: Information Sources and Search Strategy; Supplementary Methods S1.2: Retrospectively Reconstructed, Reproducibility-Oriented Search Strategies; Supplementary Methods S1.3: Record Management and Study Selection; Supplementary Methods S1.4: Direct Evidence, Zero Events, and Trial Families; Supplementary Methods S1.5: Participant-Level Clustering; Supplementary Methods S1.6: Adjusted Time-to-Event Effects; Supplementary Methods S1.7: Exploratory Network and Inconsistency; Supplementary Methods S1.8: Certainty and Reporting-Bias Assessment; Table S1: Recorded database search dates and yields; Table S2: Report-level selection accounting; Table S3: Mapping of randomized trial families, reports, follow-up assessments, and outcomes; Table S4: Outcome definitions, harmonisation rules, and study-specific analytical decisions; Table S5: Arm-level outcome data used in binary analyses and descriptive long-term summaries; Table S6: Direct-evidence results under the primary working ICC of 0.10; Table S7: Adjusted time-to-event estimates retained in their published effect metric; Table S8: Sensitivity of direct analyses to the working intraclass correlation coefficient (ICC); Table S9: Rare-event, double-zero, and conditional sensitivity analyses; Table S10: Distribution of potential effect modifiers across direct contrasts in the exploratory 12–24-month late pulpal-failure network; Table S11: Exploratory consistency, unrelated mean-effects, and inconsistency analyses for late pulpal failure at 12–24 months; Table S12: Differences between the registered protocol (PROSPERO) and the methods used in this review; Table S13: GRADE evidence profile for the primary direct-evidence estimates; Table S14: GRADE assessment of adjusted time-to-event estimates; Table S15: Manual CINeMA confidence assessment for the exploratory 12–24-month late pulpal-failure network; Figure S1: Adjusted time-to-event estimates were reported separately and not pooled. Filled squares indicate primary direct estimates; the open marker indicates the Recchi secondary sensitivity estimate. Estimates from repeated Maltz-family reports are not independent confirmation. Figure S2: Outcome-specific network geometry for late pulpal failure at 12–24 months. Node size is proportional to the number of analyzed teeth in the RR-informative set; edge width and labels reflect independent trial families. Rando-Meirelles 2013 was double-zero and did not inform the RR network. The network was exploratory and was not used for ranking [47]. The supplementary evidence tables and figures cite References [18,19,40,41,42,43,44,45,46,47,48], and the computational archive is described in Reference [54]. Additional supporting files comprise the completed PRISMA 2020 checklist [31], the completed PRISMA-NMA checklist [32], and the excluded-studies workbook, which documents the reasons for excluding the reports cited in References [17,39,55,56,57,58,59,60,61,62,63,64,65].

## Abbreviations

CCR	Complete Carious Tissue Removal
SCR	Selective Carious Tissue Removal
SWR	Stepwise Carious Tissue Removal
RCT	Randomized Clinical Trial
PRISMA	Preferred Reporting Items for Systematic Reviews and Meta-Analyses
PRISMA-NMA	Preferred Reporting Items for Systematic Reviews and Meta-Analyses for Network Meta-Analyses
PROSPERO	International Prospective Register of Systematic Reviews
PICO	Population, Intervention, Comparator, Outcome
RR	Risk Ratio
95% CI	95% Confidence Interval
RoB 2	Risk of Bias 2
FDI	Fédération Dentaire Internationale / World Dental Federation
NMA	Network Meta-Analysis
REML	Restricted Maximum Likelihood
QE	Test statistic for residual heterogeneity
HR	Hazard Ratio
ICC	Intraclass Correlation Coefficient
RD	Risk Difference
UME	Unrelated Mean Effects
HK	Hartung–Knapp
GRADE	Grading of Recommendations Assessment, Development and Evaluation
CINeMA	Confidence in Network Meta-Analysis

## References

[1] Takahashi N., Nyvad B. (2011). The role of bacteria in the caries process: Ecological perspectives. J. Dent. Res..

[2] Warreth A. (2023). Dental caries and its management. Int. J. Dent..

[3] Benzian H., Beltrán-Aguilar E. (2025). The global burden of oral diseases: Stronger data for stronger action. Lancet.

[4] World Health Organization (2022). Global Oral Health Status Report: Towards Universal Health Coverage for Oral Health by 2030.

[5] Black G.V. (1908). A Work on Operative Dentistry: The Technical Procedures in Filling Teeth.

[6] Mount G.J. (2001). An Atlas of Glass-Ionomer Cements.

[7] Fusayama T. (1979). Two layers of carious dentin; diagnosis and treatment. Oper. Dent..

[8] Massara M.L.A., Alves J.B., Brandão P.R.G. (2002). Atraumatic restorative treatment: Clinical, ultrastructural and chemical analysis. Caries Res..

[9] Tyas M.J., Anusavice K.J., Frencken J.E., Mount G.J. (2000). Minimal intervention dentistry: A review. Int. Dent. J..

[10] Kidd E.A.M. (2004). How clean must a cavity be before restoration?. Caries Res..

[11] Banerjee A., Watson T.F., Kidd E.A.M. (2000). Dentine caries excavation: A review of current clinical techniques. Br. Dent. J..

[12] Dammaschke T. (2026). On the necessity of complete caries excavation: A narrative review. Head Face Med..

[13] Philip N., Suneja B. (2023). The revolutionary evolution in carious lesion management. J. Conserv. Dent..

[14] Schwendicke F., Kosan E., Banerjee A., Baysan A., Bjørndal L., Ceballos L., Duncan H.F., Herbst S., Neuhaus K.W., O’COnnell A.C. (2026). Deep caries management: EFCD-ESE-ORCA S3-level clinical practice guideline. Int. Endod. J..

[15] Leksell E., Ridell K., Cvek M., Mejàre I. (1996). Pulp exposure after stepwise versus direct complete excavation of deep carious lesions in young posterior permanent teeth. Endod. Dent. Traumatol..

[16] Innes N.P.T., Frencken J.E., Bjørndal L., Maltz M., Manton D.J., Ricketts D., Van Landuyt K., Banerjee A., Campus G., Doméjean S. (2016). Managing carious lesions: Consensus recommendations on terminology. Adv. Dent. Res..

[17] Maltz M., de Oliveira E.F., Fontanella V., Bianchi R. (2002). A clinical, microbiologic, and radiographic study of deep caries lesions after incomplete caries removal. Quintessence Int..

[18] Maltz M., Koppe B., Jardim J.J., Alves L.S., de Paula L.M., Yamaguti P.M., Almeida J.C.F., Moura M.S., Mestrinho H.D. (2018). Partial caries removal in deep caries lesions: A 5-year multicenter randomized controlled trial. Clin. Oral. Investig..

[19] Bjørndal L., Reit C., Bruun G., Markvart M., Kjældgaard M., Näsman P., Thordrup M., Dige I., Nyvad B., Fransson H. (2010). Treatment of deep caries lesions in adults: Randomized clinical trials comparing stepwise versus direct complete excavation, and direct pulp capping versus partial pulpotomy. Eur. J. Oral. Sci..

[20] Dhar V., Pilcher L., Fontana M., González-Cabezas C., Keels M.A., Mascarenhas A.K., Nascimento M., Platt J.A., Sabino G.J., Slayton R. (2023). Evidence-based clinical practice guideline on restorative treatments for caries lesions: A report from the American Dental Association. J. Am. Dent. Assoc..

[21] Duncan H.F., Galler K.M., Tomson P.L., Simon S., El-Karim I., Kundzina R., Krastl G., Dammaschke T., Fransson H., Markvart M. (2019). European Society of Endodontology position statement: Management of deep caries and the exposed pulp. Int. Endod. J..

[22] Landys Borén D., Jonasson P., Kvist T. (2015). Long-term survival of endodontically treated teeth at a public dental specialist clinic. J. Endod..

[23] Duncan H.F., Kirkevang L.L., Peters O.A., El-Karim I., Krastl G., Del Fabbro M., Chong B.S., Galler K.M., Segura-Egea J.J., Kebschull M. (2023). Treatment of pulpal and apical disease: The European Society of Endodontology (ESE) S3-level clinical practice guideline. Int. Endod. J..

[24] Wang W., Zeng Q., Li Y., Sun Y., Kim T., Tang J., Bergeron B.E., Tay F., Gu L. (2024). Effectiveness of pulpotomy in managing carious exposure in mature permanent teeth: A systematic review and meta-analysis. J. Dent..

[25] Figundio N., Lopes P., Tedesco T.K., Fernandes J.C.H., Fernandes G.V.O., Mello-Moura A.C.V. (2023). Deep carious lesions management with stepwise, selective, or non-selective removal in permanent dentition: A systematic review of randomized clinical trials. Healthcare.

[26] Schwendicke F., Walsh T., Lamont T., Al-Yaseen W., Bjørndal L., Clarkson J.E., Fontana M., Rossi J.G., Göstemeyer G., Levey C. (2021). Interventions for treating cavitated or dentine carious lesions. Cochrane Database Syst. Rev..

[27] Schwendicke F., Dörfer C.E., Paris S. (2013). Incomplete caries removal: A systematic review and meta-analysis. J. Dent. Res..

[28] Li T., Zhai X., Song F., Zhu H. (2018). Selective versus non-selective removal for dental caries: A systematic review and meta-analysis. Acta Odontol. Scand..

[29] Yao Y., Luo A., Hao Y. (2023). Selective versus stepwise removal of deep carious lesions: A meta-analysis of randomized controlled trials. J. Dent. Sci..

[30] Ramezanzade S., Bjørndal L., Chen H., Baysan A. (2026). Effectiveness of stepwise excavation or selective excavation in comparison with non-selective caries removal in managing deep caries in vital permanent teeth: A systematic review with trial sequential, pairwise, and network meta-analyses. Caries Res..

[31] Page M.J., McKenzie J.E., Bossuyt P.M., Boutron I., Hoffmann T.C., Mulrow C.D., Shamseer L., Tetzlaff J.M., Akl E.A., Brennan S.E. (2021). The PRISMA 2020 statement: An updated guideline for reporting systematic reviews. BMJ.

[32] Hutton B., Salanti G., Caldwell D.M., Chaimani A., Schmid C.H., Cameron C., Ioannidis J.P.A., Straus S., Thorlund K., Jansen J.P. (2015). The PRISMA extension statement for reporting of systematic reviews incorporating network meta-analyses of health care interventions: Checklist and explanations. Ann. Intern. Med..

[33] Ouzzani M., Hammady H., Fedorowicz Z., Elmagarmid A. (2016). Rayyan: A web and mobile app for systematic reviews. Syst. Rev..

[34] Guimarães N.S., Ferreira A.J.F., Silva R.C.R., de Paula A.A., Lisboa C.S., Magno L., Ichiara M.Y., Barreto M.L. (2022). Deduplicating records in systematic reviews: There are free, accurate automated ways to do so. J. Clin. Epidemiol..

[35] Valizadeh A., Moassefi M., Nakhostin-Ansari A., Hosseini Asl S.H., Saghab Torbati M., Aghajani R., Ghorbani Z.M., Faghani S. (2022). Abstract screening using the automated tool Rayyan: Results of effectiveness in three diagnostic test accuracy systematic reviews. BMC Med. Res. Methodol..

[36] Sterne J.A.C., Savović J., Page M.J., Elbers R.G., Blencowe N.S., Boutron I., Cates C.J., Cheng H.Y., Corbett M.S., Eldridge S.M. (2019). RoB 2: A revised tool for assessing risk of bias in randomised trials. BMJ.

[37] Guyatt G.H., Oxman A.D., Vist G.E., Kunz R., Falck-Ytter Y., Alonso-Coello P., Schünemann H.J. (2008). GRADE: An emerging consensus on rating quality of evidence and strength of recommendations. BMJ.

[38] Nikolakopoulou A., Higgins J.P.T., Papakonstantinou T., Chaimani A., Del Giovane C., Egger M., Salanti G. (2020). CINeMA: An approach for assessing confidence in the results of a network meta-analysis. PLoS Med..

[39] Orhan A.I., Oz F.T., Orhan K. (2010). Pulp exposure occurrence and outcomes after 1- or 2-visit indirect pulp therapy vs. complete caries removal in primary and permanent molars. Pediatr. Dent..

[40] Ahmed M.R., Aaslam S., Bukhari J.H. (2021). Comparison of partial and complete caries excavation in permanent teeth: An 18 months follow-up. Pak. J. Med. Health Sci..

[41] Bjørndal L., Fransson H., Bruun G., Markvart M., Kjældgaard M., Näsman P., Hedenbjörk-Lager A., Dige I., Thordrup M. (2017). Randomized clinical trials on deep carious lesions: 5-year follow-up. J. Dent. Res..

[42] Khokhar M., Tewari S. (2018). Outcomes of partial and complete caries excavation in permanent teeth: A 18 month clinical study. Contemp. Clin. Dent..

[43] Labib M.E., Hassanein O.E., Moussa M., Yassen A., Schwendicke F. (2019). Selective versus stepwise removal of deep carious lesions in permanent teeth: A randomised controlled trial from Egypt—An interim analysis. BMJ Open.

[44] Maltz M., Jardim J.J., Mestrinho H.D., Yamaguti P.M., Podestá K., Moura M.S., de Paula L. (2013). Partial removal of carious dentine: A multicenter randomized controlled trial and 18-month follow-up results. Caries Res..

[45] Maltz M., Garcia R., Jardim J.J., de Paula L.M., Yamaguti P.M., Moura M.S., Garcia F., Nascimento C., Oliveira A., Mestrinho H. (2012). Randomized trial of partial versus stepwise caries removal: 3-year follow-up. J. Dent. Res..

[46] Jardim J.J., Mestrinho H.D., Koppe B., de Paula L.M., Alves L.S., Yamaguti P.M., Almeida J.C.F., Maltz M. (2020). Restorations after selective caries removal: 5-year randomized trial. J. Dent..

[47] Rando-Meirelles M.P.M., Torres L.H.D.N., de Sousa M.d.L.R. (2013). Twenty-four months of follow-up after partial removal of carious dentin: A preliminary study. Dentistry.

[48] Recchi A.F., Azambuja R.S.D., Alves L.S., Maltz M., Jardim J.J. (2024). Restorations performance after selective caries removal to soft dentine: 18-month follow-up of a controlled clinical trial. J. Dent..

[49] Smith A.J., Murray P.E., Sloan A.J., Matthews J.B., Zhao S. (2001). Trans-dentinal stimulation of tertiary dentinogenesis. Adv. Dent. Res..

[50] Bjørndal L., Larsen T. (2000). Changes in the cultivable flora in deep carious lesions following a stepwise excavation procedure. Caries Res..

[51] Paddick J.S., Brailsford S.R., Kidd E.A.M., Beighton D. (2005). Phenotypic and genotypic selection of microbiota surviving under dental restorations. Appl. Environ. Microbiol..

[52] Marsh P.D. (2010). Microbiology of dental plaque biofilms and their role in oral health and caries. Dent. Clin. N. Am..

[53] Bitello-Firmino L., Soares V.K., Damé-Teixeira N., Parolo C.C.F., Maltz M. (2018). Microbial load after selective and complete caries removal in permanent molars: A randomized clinical trial. Braz. Dent. J..

[54] Mascarenhas P. (2026). R Script, Dataset and Output [Data Set].

[55] Maltz M., Oliveira E.F., Fontanella V., Carminatti G. (2007). Deep caries lesions after incomplete dentine caries removal: 40-month follow-up study. Caries Res..

[56] Casagrande L., Seminario A.T., Correa M.B., Werle S.B., Maltz M., Demarco F.F., de Araujo F.B. (2017). Longevity and associated risk factors in adhesive restorations of young permanent teeth after complete and selective caries removal: A retrospective study. Clin. Oral Investig..

[57] Oz F.D., Bolay S., Bayazit E.O., Bicer C.O., Isikhan S.Y. (2019). Long-term survival of different deep dentin caries treatments: A 5-year clinical study. Niger. J. Clin. Pract..

[58] Ortega-Verdugo P., Guzmán-Armstrong S., Cobb D., Dawson D.V., Blanchette D., Kolker J.L., Hernández M., Warren J.J. (2016). Factors associated with reevaluation of the stepwise excavation procedure: An 8-year retrospective study. Caries Res..

[59] Melgar X.C., Opdam N.J.M., Britto Correa M., Franzon R., Demarco F.F., Araujo F.B., Casagrande L. (2017). Survival and associated risk factors of selective caries removal treatments in primary teeth: A retrospective study in a high caries risk population. Caries Res..

[60] Vaghasiya J., Mittal S., Choudhari S.R., Rishitha N. (2024). Complete versus incomplete caries removal procedures and their effects on dental pulp in primary teeth—An in vivo study. J. Indian Soc. Pedod. Prev. Dent..

[61] Elhennawy K., Finke C., Paris S., Reda S., Jost-Brinkmann P.-G., Schwendicke F. (2021). Selective vs stepwise removal of deep carious lesions in primary molars: 24 months follow-up from a randomized controlled trial. Clin. Oral Investig..

[62] Liberman J., Franzon R., Guimarães L.F., Casagrande L., Haas A.N., Araujo F.B. (2020). Survival of composite restorations after selective or total caries removal in primary teeth and predictors of failures: A 36-months randomized controlled trial. J. Dent..

[63] Franzon R., Guimarães L.F., Magalhães C.E., Haas A.N., Araujo F.B. (2014). Outcomes of one-step incomplete and complete excavation in primary teeth: A 24-month randomized controlled trial. Caries Res..

[64] Lula E.C.O., Monteiro-Neto V., Alves C.M.C., Ribeiro C.C.C. (2009). Microbiological analysis after complete or partial removal of carious dentin in primary teeth: A randomized clinical trial. Caries Res..

[65] Foley J., Evans D., Blackwell A. (2004). Partial caries removal and cariostatic materials in carious primary molar teeth: A randomised controlled clinical trial. Br. Dent. J..

