# Supplementary material for: Complete, Selective, and Stepwise Carious Tissue Removal for Deep Carious Lesions in Vital Permanent Teeth: A Systematic Review and Meta-Analysis
Source: Dent J (Basel). 2026 Sep 20;14(9):610. doi: 10.3390/dj14090610 (PMC13605839; doi:10.3390/dj14090610)
Supplement: Supplementary file 1 [file dentistry-14-00610-s001.zip › PRISMA_NMA_Checklist.pdf]

PRISMA-NMA Checklist - Completed

Manuscript Version 3.0 | Supplementary Material Version 3.0 | Location audit: 1 September 2026

From: Hutton B, Salanti G, Caldwell DM, et al. The PRISMA Extension Statement for Reporting of Systematic Reviews Incorporating Network Meta-analyses of Health Care Interventions: Checklist and Explanations. Ann Intern Med. 2015;162(11):777-784. doi:10.7326/M14-2385.

Audit legend: Green = reported; Yellow = partially reported or pending final verification; Red = not reported / requires corrective action. The 2015 PRISMA-NMA extension is applied alongside PRISMA 2020. Page references were verified against the rendered 20-page manuscript and 18-page supplement.

The strength-of-evidence item is fulfilled: GRADE methods/results are reported in the manuscript and Supplementary Tables S13–S14, and CINeMA confidence is reported for all three exploratory network estimates in Supplementary Table S15. Remaining partial items are: the title does not explicitly identify a network meta-analysis (Item 1); the abstract does not describe the network-analysis method or report a network estimate (Item 2); the retrospectively reconstructed search histories cannot fully reproduce unavailable historical platform filters and citation-tracking details (Item 8); and no investigator-contact process was reported (Item 10).

Section / Topic	Item #	Checklist item	Location where item is reported
**TITLE**			
Title	1	Identify the report as a systematic review incorporating a network meta-analysis (or related form of meta-analysis).	**Partially reported –** Main manuscript p. 1 (title identifies a systematic review and meta-analysis but does not explicitly identify the network meta-analysis; the network is described as exploratory in the report).
**ABSTRACT**			
Structured summary	2	Provide a structured summary including, as applicable: background/objectives; data sources; eligibility criteria, participants and interventions; study appraisal; synthesis methods including network meta-analysis; number of studies and participants; summary estimates with confidence/credible intervals; limitations; conclusions and implications; primary funding source; and registration information.	**Partially reported –** Main manuscript p. 1 (structured abstract reports sources, eligibility, RoB 2, direct synthesis, included reports/families, pooled direct estimates, certainty, limitations, funding, and registration, but no NMA-specific method or network estimate).
**INTRODUCTION**			
Rationale	3	Describe the rationale for the review in the context of what is already known, including why a network meta-analysis has been conducted.	**Reported –** Main manuscript pp. 1–2.
Objectives	4	Provide an explicit statement of the questions being addressed, with reference to participants, interventions, comparisons, outcomes, and study design (PICOS).	**Reported –** Main manuscript p. 2.
**METHODS**			
Protocol and registration	5	Indicate whether a review protocol exists and, if so, where it can be accessed; provide registration information, including registration number.	**Reported –** Main manuscript p. 2; Supplementary material p. 15, Table S12.
Eligibility criteria	6	Specify study and report characteristics used as eligibility criteria, giving rationale. Clearly describe eligible treatments in the network and whether any treatments were clustered or merged into the same node, with justification.	**Reported –** Main manuscript pp. 2–3.
Information sources	7	Describe all information sources, including databases and dates of coverage, contact with study authors if used, and date last searched.	**Reported –** Main manuscript p. 3; Supplementary material p. 1.
Search	8	Present the full electronic search strategy for at least one database, including any limits used, so that it could be repeated.	**Partially reported –** Main manuscript p. 3; Supplementary material p. 1 (database-specific strategies are retrospective reconstructions; exact saved interface syntax/filters and citation-tracking date/yield are unavailable).
Study selection	9	State the process for selecting studies, including screening, eligibility, inclusion in the systematic review, and inclusion in meta-analysis where applicable.	**Reported –** Main manuscript p. 3 and pp. 5–6, Figure 1; Supplementary material p. 2.

Section / Topic	Item #	Checklist item	Location where item is reported
Data collection process	10	Describe the method of data extraction from reports, including piloted forms, independent duplicate extraction, and any processes for obtaining or confirming data from investigators.	**Partially reported –** Main manuscript pp. 3–4 (piloted duplicate extraction and adjudication are described; no investigator-contact/confirmation process is documented).
Data items	11	List and define all variables for which data were sought, including PICOS, and describe assumptions and simplifications.	**Reported –** Main manuscript pp. 3–4 and Table 1 on pp. 7–8; Supplementary material pp. 3–8.
Geometry of the network	S1	Describe methods used to explore the geometry of the treatment network and potential biases related to it, including graphical presentation and characteristics compiled to describe the evidence base.	**Reported –** Main manuscript p. 5; Supplementary material pp. 12–14, Table S10, Figure S2, and Table S11.
Risk of bias within individual studies	12	Describe methods for assessing risk of bias of individual studies, including whether assessment was at study or outcome level, and how the information was used in synthesis.	**Reported –** Main manuscript p. 4.
Summary measures	13	State the principal summary measures and any additional measures such as treatment rankings or SUCRA values, including modified approaches used to present findings.	**Reported –** Main manuscript pp. 4–5.
Planned methods of analysis	14	Describe methods of handling data and combining study results for each network meta-analysis, including handling of multi-arm trials, variance structure, prior distributions for Bayesian analyses, and assessment of model fit.	**Reported –** Main manuscript pp. 4–5; Supplementary material pp. 2–3 and 13–14 (frequentist variance structure, consistency/UME and inconsistency models; no multi-arm trials and no Bayesian priors).
Assessment of inconsistency	S2	Describe statistical methods used to evaluate agreement of direct and indirect evidence and efforts to address inconsistency if present.	**Reported –** Main manuscript p. 5; Supplementary material pp. 13–14, Table S11.
Risk of bias across studies	15	Specify any assessment of risk of bias that may affect the cumulative evidence, such as publication bias or selective reporting.	**Reported –** Main manuscript p. 5; Supplementary material pp. 3, 16 and 18, Tables S13 and S15.
Additional analyses	16	Describe methods of additional analyses, indicating which were prespecified, such as sensitivity analyses, subgroup/meta-regression analyses, alternative network formulations, or alternative prior distributions.	**Reported –** Main manuscript pp. 4–5; Supplementary material pp. 2–3 and 10–15.
**RESULTS**			
Study selection	17	Give numbers of studies screened, assessed for eligibility, and included, with reasons for exclusions, ideally using a flow diagram.	**Reported –** Main manuscript pp. 5–6, Figure 1; Supplementary material p. 2, Table S2. Eleven reports from seven randomized trial families were included.
Presentation of network structure	S3	Provide a network graph of the included studies to enable visualization of treatment-network geometry.	**Reported –** Supplementary material p. 13, Figure S2; referenced in Main manuscript pp. 12–13.
Summary of network geometry	S4	Provide a brief overview of treatment-network characteristics, including abundance of trials/patients for interventions and comparisons, evidence gaps, and potential biases reflected by network structure.	**Reported –** Main manuscript pp. 12–13; Supplementary material p. 12, Table S10, and pp. 13–14, Figure S2 and Table S11.
Study characteristics	18	For each study, present extracted characteristics such as study size, PICOS, and follow-up, and provide citations.	**Reported –** Main manuscript pp. 6–8, Table 1; Supplementary material p. 3, Table S3.
Risk of bias within studies	19	Present risk-of-bias data for each study and, where available, outcome-level assessments.	**Reported –** Main manuscript pp. 8–9, Figure 2 (outcome-specific RoB 2 judgments for all 11 reports).
Results of individual studies	20	For all outcomes, present per-study group summary data and effect estimates with confidence intervals, using modified presentation approaches where needed for networks.	**Reported –** Main manuscript pp. 7–12, Tables 1–3 and Figures 2–3; Supplementary material pp. 5–12.
Synthesis of results	21	Present results of each meta-analysis with confidence/credible intervals; consider league tables or forest plots and present additional summary measures if explored.	**Reported –** Main manuscript pp. 9–14, Tables 2–4 and Figure 3; Supplementary material pp. 7–14. No rankings or league table were generated.
Exploration for inconsistency	S5	Describe results from investigations of inconsistency, including model-fit comparisons, P values, or inconsistency estimates from different parts of the network.	**Reported –** Main manuscript pp. 12–13; Supplementary material pp. 13–14, Table S11.
Risk of bias across studies	22	Present results of any assessment of risk of bias across studies for the evidence base.	**Reported –** Main manuscript p. 5 and pp. 15–16; Supplementary material pp. 16 and 18, Tables S13 and S15.
Results of additional analyses	23	Give results of additional analyses, such as sensitivity analyses, subgroup/meta-regression analyses, alternative network geometries, or alternative priors.	**Reported –** Main manuscript pp. 10–13; Supplementary material pp. 10–14.

Section / Topic	Item #	Checklist item	Location where item is reported
**DISCUSSION**			
Summary of evidence	24	Summarize main findings, including the strength of evidence for each main outcome, and consider relevance to clinicians, patients, and policy-makers.	**Reported –** Main manuscript pp. 13–16, Table 4, Discussion, Clinical Implications, and Conclusions; Supplementary material pp. 16–18, Tables S13–S15.
Limitations	25	Discuss limitations at study/outcome and review levels, and comment on validity of transitivity and consistency assumptions and concerns regarding network geometry.	**Reported –** Main manuscript pp. 14–16; Supplementary material pp. 12–14.
Conclusions	26	Provide a general interpretation in the context of other evidence and implications for future research.	**Reported –** Main manuscript pp. 14–16.
**FUNDING**			
Funding	27	Describe funding and other support, the role of funders, and relevant manufacturer funding or professional conflicts that could affect use of treatments in the network.	**Reported –** Main manuscript pp. 16–17 (funding, funder role, and conflicts-of-interest statements).
