# Supplementary material for: Complete, Selective, and Stepwise Carious Tissue Removal for Deep Carious Lesions in Vital Permanent Teeth: A Systematic Review and Meta-Analysis
Source: Dent J (Basel). 2026 Sep 20;14(9):610. doi: 10.3390/dj14090610 (PMC13605839; doi:10.3390/dj14090610)
Supplement: Supplementary file 1 [file dentistry-14-00610-s001.zip › Supplementary_Material_ref.pdf]

Supplementary Materials

Complete, Selective, and Stepwise Carious Tissue Removal for Deep Carious Lesions in Vital Permanent Teeth: A Systematic Review and Meta-Analysis

S1. Supplementary Methods

S1.1. Information Sources and Search Strategy

The searches covered PubMed/MEDLINE, Embase, Scopus, the Cochrane Library, and B-on from inception to 20 January 2026. PubMed/MEDLINE and Embase provided complementary biomedical and dental indexing; Scopus added multidisciplinary citation coverage; the Cochrane Library focused on controlled trial and review records; and B-on served as a Portuguese discovery and access service across subscribed collections. Backward and forward citation tracking of included reports supplemented these sources; its date and yield were not separately recorded.

Table S1. Recorded database search dates and yields.

Database / interface	Search date	Recorded yield
PubMed/MEDLINE	20 January 2026	147
Scopus	20 January 2026	55
Embase	20 January 2026	287
Cochrane Library	20 January 2026	32
B-on discovery service	20 January 2026	509
Total before deduplication	20 January 2026	1,030

S1.2. Retrospectively Reconstructed, Reproducibility-Oriented Search Strategies

Native platform histories were not archived. The database-specific strings below are retrospective, reproducibility-oriented reconstructions based on the preserved concept strategy and applicable platform syntax; they were not rerun and are not presented as verbatim historical searches. The yields recorded in Table S1 refer to the original searches; exact interface-specific filters cannot be verified.

PubMed/MEDLINE (Title/Abstract): (("deep caries lesion"[Title/Abstract] OR "deep cavities"[Title/Abstract] OR "profound carious lesions"[Title/Abstract] OR "deep caries"[Title/Abstract]) AND (("partial caries excavation"[Title/Abstract] OR "selective caries removal"[Title/Abstract] OR "selective excavation"[Title/Abstract]) OR ("complete caries excavation"[Title/Abstract] OR "non-selective caries removal"[Title/Abstract] OR "total caries removal"[Title/Abstract]) OR ("stepwise excavation"[Title/Abstract])))

Scopus: TITLE-ABS-KEY(("deep caries lesion" OR "deep cavities" OR "profound carious lesions" OR "deep caries") AND (("partial caries excavation" OR "selective caries removal" OR "selective excavation") OR ("complete caries excavation" OR "non-selective caries removal" OR "total caries removal") OR ("stepwise excavation")))

Embase: (('deep caries lesion':ti,ab,kw OR 'deep cavities':ti,ab,kw OR 'profound carious lesions':ti,ab,kw OR 'deep caries':ti,ab,kw) AND (('partial caries excavation':ti,ab,kw OR 'selective caries removal':ti,ab,kw OR 'selective excavation':ti,ab,kw) OR ('complete caries excavation':ti,ab,kw OR 'non-selective caries removal':ti,ab,kw OR 'total caries removal':ti,ab,kw) OR ('stepwise excavation':ti,ab,kw)))

Cochrane Library: (("deep caries lesion":ti,ab,kw OR "deep cavities":ti,ab,kw OR "profound carious lesions":ti,ab,kw OR "deep caries":ti,ab,kw) AND (("partial caries excavation":ti,ab,kw OR "selective caries removal":ti,ab,kw OR "selective excavation":ti,ab,kw) OR ("complete caries excavation":ti,ab,kw OR "non-selective caries removal":ti,ab,kw OR "total caries removal":ti,ab,kw) OR ("stepwise excavation":ti,ab,kw)))

B-on (all fields): ("deep caries lesion" OR "deep cavities" OR "profound carious lesions" OR "deep caries") AND (("partial caries excavation" OR "selective caries removal" OR "selective excavation") OR ("complete caries excavation" OR "non-selective caries removal" OR "total caries removal") OR ("stepwise excavation"))

S1.3. Record Management and Study Selection

Search results were imported into Rayyan. Candidate duplicates flagged by the software were manually reviewed before deletion. Two reviewers independently screened titles/abstracts and full texts; automated predictions did not replace human eligibility decisions. Disagreements were resolved through discussion or by a third reviewer. Reports not retrieved were kept separate from excluded full texts.

Table S2. Report-level selection accounting.

Assessment status	Number	Reason/category	Analytical disposition
Reports sought for retrieval	28	Potentially eligible after title/abstract screening	Four were not retrieved
Reports not retrieved	4	Full text unavailable	Eligibility could not be confirmed
Full-text reports assessed	24	Evaluated against eligibility criteria	Thirteen excluded; eleven included
Excluded full-text reports	6	Non-randomized design	Excluded
Excluded full-text reports	6	Primary dentition only	Excluded
Excluded full-text report	1	Wrong population: Orhan et al. (2010)	Mixed primary molars and young permanent first molars; in the permanent-tooth subset, root maturity was not reported by arm and no compatible subgroup was separately extractable.
Reports included	11	Eligible randomized reports	Seven independent randomized trial families

Note: The excluded-studies workbook lists all 13 excluded full-text reports, each with a single primary reason: six non-randomized reports, six primary-dentition reports, and Orhan et al. (2010) [39]. Orhan included primary molars and young permanent first molars; in the potentially eligible permanent-tooth subset, root maturity was not reported by treatment arm, and no subgroup compatible with the post-registration scope clarification was separately extractable. It contributes no data to extraction, risk-of-bias assessment, GRADE, figures, direct analyses, or the exploratory network.

S1.4. Direct Evidence, Zero Events, and Trial Families

Binary outcomes were analyzed as RRs with 95% CIs. A 0.5 continuity correction was applied only when a comparison contained a zero cell. Double-zero comparisons provide no information on relative effects and were retained in event tables and Newcombe-Wilson risk-difference sensitivities. One estimate per trial family, outcome, follow-up window, and contrast was included in the synthesis. Pooling required at least three informative independent families and used REML with modified Knapp-Hartung inference; the safeguard prevented a CI narrower than the corresponding Wald CI.

S1.5. Participant-Level Clustering

When more than one tooth per participant was possible and no adjusted estimate was available, the sampling variance was multiplied by $1 + (m - 1) \times ICC$, where m is the mean number of analyzed teeth per participant for the relevant report and outcome. No empirical intraclass correlation coefficient (ICC) was reported. ICC = 0.10 represented a modest positive correlation; values of 0, 0.05, and 0.20 indicated independence, low clustering, and moderate clustering, respectively. The design effect alters precision, not arm-level event risks.

S1.6. Adjusted Time-to-Event Effects

Published adjusted HRs were retained in their reported time-to-event metric and neither converted to binary-effect estimates nor pooled with RRs. Estimates from different outcomes, windows, or repeated reports of the same family were not treated as independent confirmation. Conditional late outcomes that excluded intraoperative exposures after randomization were considered sensitivity evidence only.

S1.7. Exploratory Network and Inconsistency

The supplementary contrast-based network was restricted to RR-informative 12-24-month late pulpal failure. CCR served as the reference, and a common between-family heterogeneity variance was estimated by REML. Transitivity was examined qualitatively across lesion depth, pulpal criteria, age, tooth type, region, follow-up, analysis population, restorative protocol, and exposure management. Consistency and unrelated mean-effects models were compared using a Wald contrast and a likelihood-ratio test under maximum likelihood. Each of the five families contributed one effect, so the sampling covariance matrix was diagonal and no within-family ϱ was required. No multi-arm covariance adjustment or Bayesian prior specification was applicable because all models were frequentist and each family contributed a single contrast. The tests had very low power; non-significance was not interpreted as evidence of consistency. No ranking was performed.

S1.8. Certainty and Reporting-Bias Assessment

GRADE assessed direct evidence for risk of bias, inconsistency, indirectness, imprecision, and publication bias. CINeMA assessed the exploratory network for within-study bias, across-study bias, indirectness, imprecision, heterogeneity, and incoherence. Formal funnel plots and regression-based small-study-effect tests were not performed because no pooled analysis contained enough independent families.

S2. Supplementary Evidence Tables and Figures

Table S3. Mapping of randomized trial families, reports, follow-up assessments, and outcomes.

Trial family	Registration	Report	Year	Direct contrast	Follow-up	Outcome contribution
Ahmed 2021	—	Ahmed et al. 2021 [40]	2021	SCR vs CCR	18 months	Procedural exposure; late pulpal failure
Bjørndal trial	—	Bjørndal et al. 2010 [19]	2010	SWR vs CCR	12 months	Procedural exposure; late pulpal failure
Bjørndal trial	—	Bjørndal et al. 2017 [41]	2017	SWR vs CCR	60 months	Adjusted late-pulpal time-to-event outcome
Khokhar 2018	—	Khokhar and Tewari 2018 [42]	2018	SCR vs CCR	18 months	Procedural exposure; conditional late failure
Labib 2019	—	Labib et al. 2019 [43]	2019	SCR vs SWR	12 months	Procedural exposure; late pulpal failure; restorative failure; adjusted composite

Trial family	Registration	Report	Year	Direct contrast	Follow-up	Outcome contribution
Maltz multicenter trial	NCT00887952	Maltz et al. 2013 [44]	2013	SCR vs SWR	18 months	Procedural exposure; late pulpal failure; restorative failure
Maltz multicenter trial	NCT00887952	Maltz et al. 2012 [45]	2012	SCR vs SWR	36 months	Adjusted late-pulpal time-to-event outcome
Maltz multicenter trial	NCT00887952	Maltz et al. 2018 [18]	2018	SCR vs SWR	60 months	Adjusted late-pulpal time-to-event outcome
Maltz multicenter trial	NCT00887952	Jardim et al. 2020 [46]	2020	SCR vs SWR	60 months	Restorative events; adjusted restorative time-to-event outcome
Rando-Meirelles 2013	—	Rando-Meirelles et al. 2013 [47]	2013	SCR vs CCR	24 months	Late pulpal failure (no irreversible pulpal or apical events reported); double-zero comparison
Recchi 2024	—	Recchi et al. 2024 [48]	2024	SCR vs SWR	18 months	Procedural exposure; late pulpal failure; restorative failure; adjusted restorative outcome

Note: Bjørndal 2010 and 2017 [19,41] are reports from one trial family. Maltz 2012, Maltz 2013, Maltz 2018, and Jardim 2020 [18,44,45,46] are reports from the same multicenter randomized cohort and were not treated as independent trials.

Table S4. Outcome definitions, harmonisation rules, and study-specific analytical decisions.

Outcome/report	Study-specific issue or definition	Harmonised analytical use	Restriction or sensitivity status
Intraoperative pulp exposure	Exposure occurring during the assigned excavation procedure.	Analyzed separately as a procedural outcome; denominator was the number of teeth entering the relevant excavation stage.	Not combined with subsequent pulpal failure.
Late pulpal failure	Irreversible pulpitis, pulp necrosis, apical pathology, endodontic treatment, or extraction for pulpal reasons after completion of the assigned procedure.	Exposure-related events were removed from the late component when separately identifiable; late events were counted once.	Time-to-event HRs were retained in their published metric and not mixed with RRs.
Restorative failure	Restoration loss, fracture, replacement, or failure according to the original trial criteria.	Analyzed separately from pulpal outcomes.	Double-zero comparisons did not contribute to RR pooling.
Overall clinical-radiographic or composite outcomes	Composite definitions reported by individual trials.	Recorded descriptively; excluded from outcome-specific syntheses when components could not be separated.	Not used as a primary outcome-specific endpoint.
Ahmed 2021 [40]	The report states 70 teeth overall, whereas the arm-level outcome tables contain 30 teeth per arm.	Used as complete-case procedural and 18-month analyses.	True ITT denominators were unavailable from the reported data.
Khokhar 2018 [42]	All 13 exposed CCR teeth were excluded from subsequent follow-up.	Exposure contributed to the primary procedural analysis.	Late outcome retained only as conditional sensitivity evidence.
Labib 2019 [43] and Maltz/Recchi families	More than one tooth could be contributed by a participant.	Sampling variances were inflated using a working design effect when no adjusted estimate was available.	Primary ICC = 0.10 represented a modest correlation; sensitivity ICC = 0, 0.05, and 0.20. The ICC affected sampling variance, not arm risks.
Rando-Meirelles 2013 [47]; Labib 2019 [43] and Maltz 2013 [44] restorative outcomes	Rando-Meirelles reported no unsatisfactory clinical or radiographic outcome in either arm at 24 months but did not provide a separate pulpal-event table; the other listed outcomes also had zero events in both arms.	Rando-Meirelles was counted as zero late pulpal failures because no irreversible pulpal or apical event was reported; all listed comparisons were retained in event tables and risk-difference sensitivities.	Explicit harmonisation assumption; double-zero comparisons did not contribute to RR pooling.

Table S5. Arm-level outcome data used in binary analyses and descriptive long-term summaries.

Report	Outcome	Window	Contrast	Arm	Events/total	Analysis population	Clustered	Conditional	Analytical status
Ahmed - 18-month report (2021) [40]	intraoperative pulp exposure	procedural	SCR vs CCR	SCR	0/30	complete-case procedural analysis	No	No	Primary direct
Ahmed - 18-month report (2021) [40]	intraoperative pulp exposure	procedural	SCR vs CCR	CCR	7/30	complete-case procedural analysis	No	No	Primary direct
Ahmed - 18-month report (2021) [40]	late pulpal failure	12-24 months	SCR vs CCR	SCR	2/30	complete-case 18-month follow-up	No	No	Primary direct
Ahmed - 18-month report (2021) [40]	late pulpal failure	12-24 months	SCR vs CCR	CCR	0/30	complete-case 18-month follow-up	No	No	Primary direct
Bjørndal - 12-month report (2010) [19]	intraoperative pulp exposure	procedural	SWR vs CCR	SWR	25/143	trial-level procedural analysis	No	No	Primary direct
Bjørndal - 12-month report (2010) [19]	intraoperative pulp exposure	procedural	SWR vs CCR	CCR	43/149	trial-level procedural analysis	No	No	Primary direct
Bjørndal - 12-month report (2010) [19]	late pulpal failure	12-24 months	SWR vs CCR	SWR	12/143	late pulpal events excluding intraoperative exposure	No	No	Primary direct
Bjørndal - 12-month report (2010) [19]	late pulpal failure	12-24 months	SWR vs CCR	CCR	13/149	late pulpal events excluding intraoperative exposure	No	No	Primary direct
Khokhar - 18-month report (2018) [42]	intraoperative pulp exposure	procedural	SCR vs CCR	SCR	0/67	procedural analysis	No	No	Primary direct
Khokhar - 18-month report (2018) [42]	intraoperative pulp exposure	procedural	SCR vs CCR	CCR	13/69	procedural analysis	No	No	Primary direct
Khokhar - 18-month report (2018) [42]	late pulpal failure	12-24 months	SCR vs CCR	SCR	5/67	per-protocol follow-up conditional on no exposure	No	Yes	Conditional sensitivity only
Khokhar - 18-month report (2018) [42]	late pulpal failure	12-24 months	SCR vs CCR	CCR	1/56	per-protocol follow-up conditional on no exposure	No	Yes	Conditional sensitivity only
Labib - 12-month report (2019) [43]	intraoperative pulp exposure	procedural	SCR vs SWR	SCR	0/66	intention-to-treat procedural analysis	Yes	No	Primary direct
Labib - 12-month report (2019) [43]	intraoperative pulp exposure	procedural	SCR vs SWR	SWR	5/66	intention-to-treat procedural analysis	Yes	No	Primary direct

Report	Outcome	Window	Contrast	Arm	Events/total	Analysis population	Clustered	Conditional	Analytical status
Labib - 12-month report (2019) [43]	late pulpal failure	12-24 months	SCR vs SWR	SCR	7/66	late pulpal complications excluding the five procedural exposures	Yes	No	Primary direct
Labib - 12-month report (2019) [43]	late pulpal failure	12-24 months	SCR vs SWR	SWR	5/66	late pulpal complications excluding the five procedural exposures	Yes	No	Primary direct
Labib - 12-month report (2019) [43]	restorative failure	12-24 months	SCR vs SWR	SCR	0/66	restorative follow-up	Yes	No	Primary direct
Labib - 12-month report (2019) [43]	restorative failure	12-24 months	SCR vs SWR	SWR	0/66	restorative follow-up	Yes	No	Primary direct
Maltz - 18-month report (2013) [44]	intraoperative pulp exposure	procedural	SCR vs SWR	SCR	0/153	procedural analysis	Yes	No	Primary direct
Maltz - 18-month report (2013) [44]	intraoperative pulp exposure	procedural	SCR vs SWR	SWR	4/146	procedural analysis	Yes	No	Primary direct
Maltz - 18-month report (2013) [44]	late pulpal failure	12-24 months	SCR vs SWR	SCR	1/112	available-case late pulpal outcome excluding immediate exposure-related endodontic treatment	Yes	No	Primary direct
Maltz - 18-month report (2013) [44]	late pulpal failure	12-24 months	SCR vs SWR	SWR	13/101	available-case late pulpal outcome excluding immediate exposure-related endodontic treatment	Yes	No	Primary direct
Maltz - 18-month report (2013) [44]	restorative failure	12-24 months	SCR vs SWR	SCR	0/112	available-case restorative outcome	Yes	No	Primary direct
Maltz - 18-month report (2013) [44]	restorative failure	12-24 months	SCR vs SWR	SWR	0/101	available-case restorative outcome	Yes	No	Primary direct
Jardim - 5-year restorative report (2020) [46]	restorative failure	60 months	SCR vs SWR	SCR	16/95	descriptive available-case event counts	Yes	No	Descriptive only
Jardim - 5-year restorative report (2020) [46]	restorative failure	60 months	SCR vs SWR	SWR	13/77	descriptive available-case event counts	Yes	No	Descriptive only
Rando-Meirelles - 24-month report (2013) [47]	late pulpal failure	12-24 months	SCR vs CCR	SCR	0/8	available-case follow-up	No	No	Primary direct

Report	Outcome	Window	Contrast	Arm	Events/total	Analysis population	Clustered	Conditional	Analytical status
Rando-Meirelles - 24-month report (2013) [47]	late pulpal failure	12-24 months	SCR vs CCR	CCR	0/8	available-case follow-up	No	No	Primary direct
Recchi - 18-month report (2024) [48]	intraoperative pulp exposure	procedural	SCR vs SWR	SCR	0/76	procedural analysis after allocation	Yes	No	Primary direct
Recchi - 18-month report (2024) [48]	intraoperative pulp exposure	procedural	SCR vs SWR	SWR	3/76	procedural analysis after allocation	Yes	No	Primary direct
Recchi - 18-month report (2024) [48]	late pulpal failure	12-24 months	SCR vs SWR	SCR	3/76	pulp necrosis by 18 months	Yes	No	Primary direct
Recchi - 18-month report (2024) [48]	late pulpal failure	12-24 months	SCR vs SWR	SWR	4/76	pulp necrosis by 18 months	Yes	No	Primary direct
Recchi - 18-month report (2024) [48]	restorative failure	12-24 months	SCR vs SWR	SCR	4/72	available-case restorative analysis	Yes	No	Primary direct
Recchi - 18-month report (2024) [48]	restorative failure	12-24 months	SCR vs SWR	SWR	2/63	available-case restorative analysis	Yes	No	Primary direct

Note: “Conditional” identifies late outcomes calculated only among teeth remaining after exclusion of intraoperative pulp exposure. Such estimates were not used in the primary late-failure synthesis.

Table S6. Direct-evidence results under the primary working ICC of 0.10.

Outcome	Contrast	Families	Events	RR (95% CI)	p value	Model	Heterogeneity / interpretation
Intraoperative pulp exposure	SCR vs SWR	3	0/295 vs 12/288	0.11 (0.003-4.64)	0.127	REML + modified Knapp-Hartung	tau-squared = 0; I-squared = 0%; QE p = 0.977. Evidence is insufficient to demonstrate a difference.
Late pulpal failure, 12-24 months	SCR vs SWR	3	11/254 vs 22/243	0.50 (0.01-21.53)	0.510	REML + modified Knapp-Hartung	tau-squared = 1.52; I-squared = 72.2%; QE p = 0.040. Extreme imprecision and heterogeneity.
Intraoperative pulp exposure	SCR vs CCR	2	Ahmed 0/30 vs 7/30; Khokhar 0/67 vs 13/69	Ahmed 0.07 (0.004-1.12); Khokhar 0.04 (0.002-0.63)	-	Study-specific only	No pooling: fewer than 3 informative families.

Outcome	Contrast	Families	Events	RR (95% CI)	p value	Model	Heterogeneity / interpretation
Intraoperative pulp exposure	SWR vs CCR	1	25/143 vs 43/149	0.61 (0.39-0.94)	0.024	Study-specific only	Single-family estimate; not pooled.
Late pulpal failure, 12-24 months	SCR vs CCR	2; 1 RR-informative	Ahmed 2/30 vs 0/30; Rando 0/8 vs 0/8	Ahmed 5.00 (0.25-99.95); Rando RR not estimable	-	Study-specific only	No pooling; one double-zero family.
Late pulpal failure, 12-24 months	SWR vs CCR	1	12/143 vs 13/149	0.96 (0.45-2.04)	0.919	Study-specific only	Single-family estimate; not pooled.
Restorative failure, 12-24 months	SCR vs SWR	3; 1 RR-informative	Recchi 4/72 vs 2/63; Labib 0/66 vs 0/66; Maltz 0/112 vs 0/101	Recchi 1.75 (0.32-9.49)	0.517	Study-specific only	No pooling; two double-zero families.

Table S7. Adjusted time-to-event estimates retained in their published effect metric.

Report	Outcome	Contrast	Months	Adjusted HR (95% CI)	Status	Analytical note
Bjørndal - 5-year report (2017) [41]	late pulpal failure	SWR vs CCR	60	0.67 (0.45–1.05)	Primary direct	Direction inverted to SWR vs CCR. Pulp exposure per se was not the late failure event in this survival model.
Labib - 12-month report (2019) [43]	overall complication	SCR vs SWR	12	0.67 (0.24–1.85)	Excluded from outcome-specific synthesis	Composite estimate combining clinically distinct outcomes; excluded from outcome-specific analyses.
Maltz - 3-year report (2012) [45]	late pulpal failure	SCR vs SWR	36	0.21 (0.07–0.63)	Primary direct	The adjusted HR was obtained from the full-text report and retained in its reported time-to-event metric; binary event counts were not used.
Maltz - 5-year report (2018) [18]	late pulpal failure	SCR vs SWR	60	0.38 (0.23–0.63)	Primary direct	Adjusted 60-month estimate reported by Maltz et al. (2018) [18].
Jardim - 5-year restorative report (2020) [46]	restorative failure	SCR vs SWR	60	0.83 (0.41–1.69)	Primary direct	Same trial family as Maltz 2012/2013/2018 [18,44,45]; adjusted restorative estimate reported by Jardim et al. (2020) [46].
Recchi - 18-month report (2024) [48]	restorative failure	SCR vs SWR	18	3.85 (0.58–25.00)	Secondary/sensitivity	Direction inverted to SCR vs SWR. The published model used restoration as the unit of analysis and did not explicitly model patient clustering.

Note: Adjusted estimates were not pooled across outcomes, follow-up windows, or repeated reports from the same family. The Labib composite estimate was recorded descriptively but excluded from the outcome-specific synthesis and analytical figure because it did not correspond to a single endpoint.

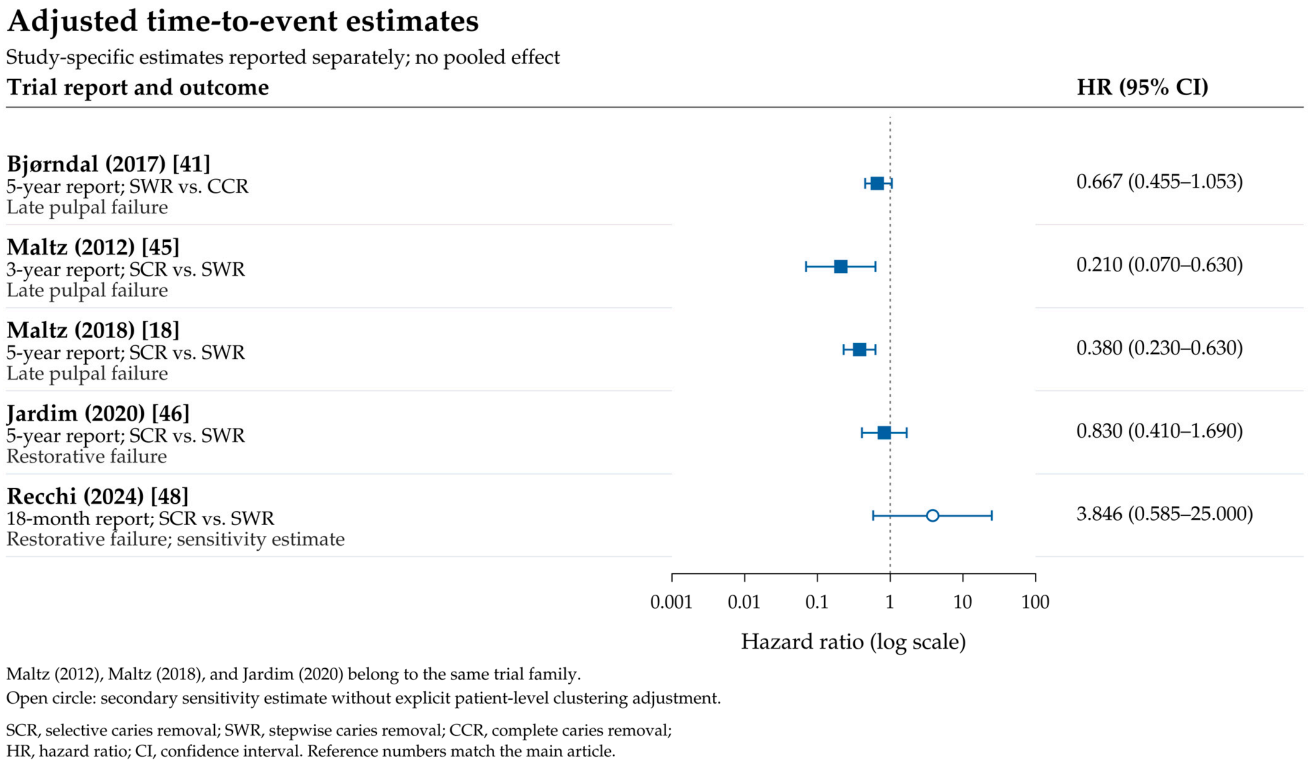

Figure S1. Adjusted time-to-event estimates were reported separately and not pooled. Filled squares indicate primary direct estimates; the open marker indicates the Recchi secondary sensitivity estimate. Estimates from repeated Maltz-family reports are not independent confirmation.

Table S8. Sensitivity of direct analyses to the working intraclass correlation coefficient (ICC).

Outcome	Contrast	ICC 0	ICC 0.05	ICC 0.10	ICC 0.20
Intraoperative pulp exposure	SCR vs SWR	0.11 (0.003-4.44)	0.11 (0.003-4.54)	0.11 (0.003-4.64)	0.11 (0.003-4.85)
Late pulpal failure, 12-24 months	SCR vs SWR	0.50 (0.01-21.52)	0.50 (0.01-21.52)	0.50 (0.01-21.53)	0.50 (0.01-21.53)
Intraoperative pulp exposure	SCR vs CCR	No pooling	No pooling	No pooling	No pooling
Intraoperative pulp exposure	SWR vs CCR	No pooling	No pooling	No pooling	No pooling
Late pulpal failure, 12-24 months	SCR vs CCR	No pooling	No pooling	No pooling	No pooling
Late pulpal failure, 12-24 months	SWR vs CCR	No pooling	No pooling	No pooling	No pooling
Restorative failure, 12-24 months	SCR vs SWR	No pooling	No pooling	No pooling	No pooling

Note: Values are pooled RRs with 95% CIs only for the two outcome-contrast analyses meeting the minimum of three informative independent families (both SCR versus SWR). Changing the ICC did not alter point estimates or conclusions, but modestly affected CI widths.

Table S9. Rare-event, double-zero, and conditional sensitivity analyses.

Analysis / report	Outcome	Contrast / metric	Events or estimate	95% CI; p value	Analytical status
Primary modified Knapp-Hartung	Intraoperative exposure	SCR vs SWR; RR	0.11	0.003-4.64; p = 0.127	Primary; continuity correction 0.5
No-correction RD model	Intraoperative exposure	SCR vs SWR; RD	-0.036	-0.083 to 0.012; p = 0.084	Sensitivity; modified Knapp-Hartung
Conventional Knapp-Hartung	Intraoperative exposure	SCR vs SWR; RR	0.11	0.063-0.196; p = 0.004	Methodological sensitivity; variance scale q = 0.023
Exact conditional CMH	Intraoperative exposure	SCR vs SWR; common OR	0.00	0.00-0.344; p < 0.001	Sensitivity only; no clustering adjustment; common-effect assumption
Rando-Meirelles 2013 [47]	Late pulpal failure	SCR vs CCR; RD	0/8 vs 0/8; RD 0.00	-0.324 to 0.324	Double-zero; excluded from RR pooling
Labib 2019 [43]	Restorative failure	SCR vs SWR; RD	0/66 vs 0/66; RD 0.00	-0.056 to 0.056	Double-zero; excluded from RR pooling
Maltz 2013 [44]	Restorative failure	SCR vs SWR; RD	0/112 vs 0/101; RD 0.00	-0.038 to 0.034	Double-zero; excluded from RR pooling
Khokhar 2018 [42]	Late pulpal failure	SCR vs CCR; RR	5/67 vs 1/56; RR 4.18	0.50-34.73; p = 0.186	Conditional on no exposure; sensitivity only

Table S10. Distribution of potential effect modifiers across direct contrasts in the exploratory 12-24-month late pulpal-failure network.

Direct contrast	Informative trial families	Follow-up	Lesion-depth criteria	Age / clinical context	Region	Outcome and analysis features	Transitivity implication
SCR vs CCR	Ahmed 2021 [40] (1)	18 months	At least 50% of dentin	Mean age 25.6 years (SCR) and 23.4 years (CCR); permanent molars	Pakistan / Asia	Composite restorations; complete-case late outcome (30/arm); seven CCR exposures managed by pulpotomy and analyzed separately	Single young-adult Asian family; post-exposure management and complete-case analysis differed from the other contrasts
SWR vs CCR	Bjørndal 2010 [19] (1)	12 months	At least 75% of dentin	Adults older than 15 years; mean age approximately 29 years; mixed permanent tooth types	Denmark and Sweden / Europe	Late pulpal failure separated from procedural exposure; calcium-hydroxide/adhesive restorative protocol	Single European family with substantially deeper lesions and a different restorative pathway
SCR vs SWR	Labib 2019 [43]; Maltz 2013 [44]; Recchi 2024 [48] (3)	12-18 months	More than two-thirds (Labib); at least 50% (Maltz and Recchi, including lesions at least 75%)	Labib: 18-47 years; Maltz 6–53 years (mean approximately 17.2; median 14); Recchi: 9-55 years (mean 29)	Egypt / Africa; Brazil / South America	GI/composite, amalgam or resin-composite, and composite protocols; clustered tooth-level data in all three reports; exposures analyzed separately	Broader age and protocol variation within the contrast; differs geographically and clinically from both CCR-based contrasts

Note: Potential effect modifiers were compared qualitatively because the network contained too few independent trial families for reliable meta-regression. The marked imbalance across contrasts reduced confidence in the transitivity assumption.

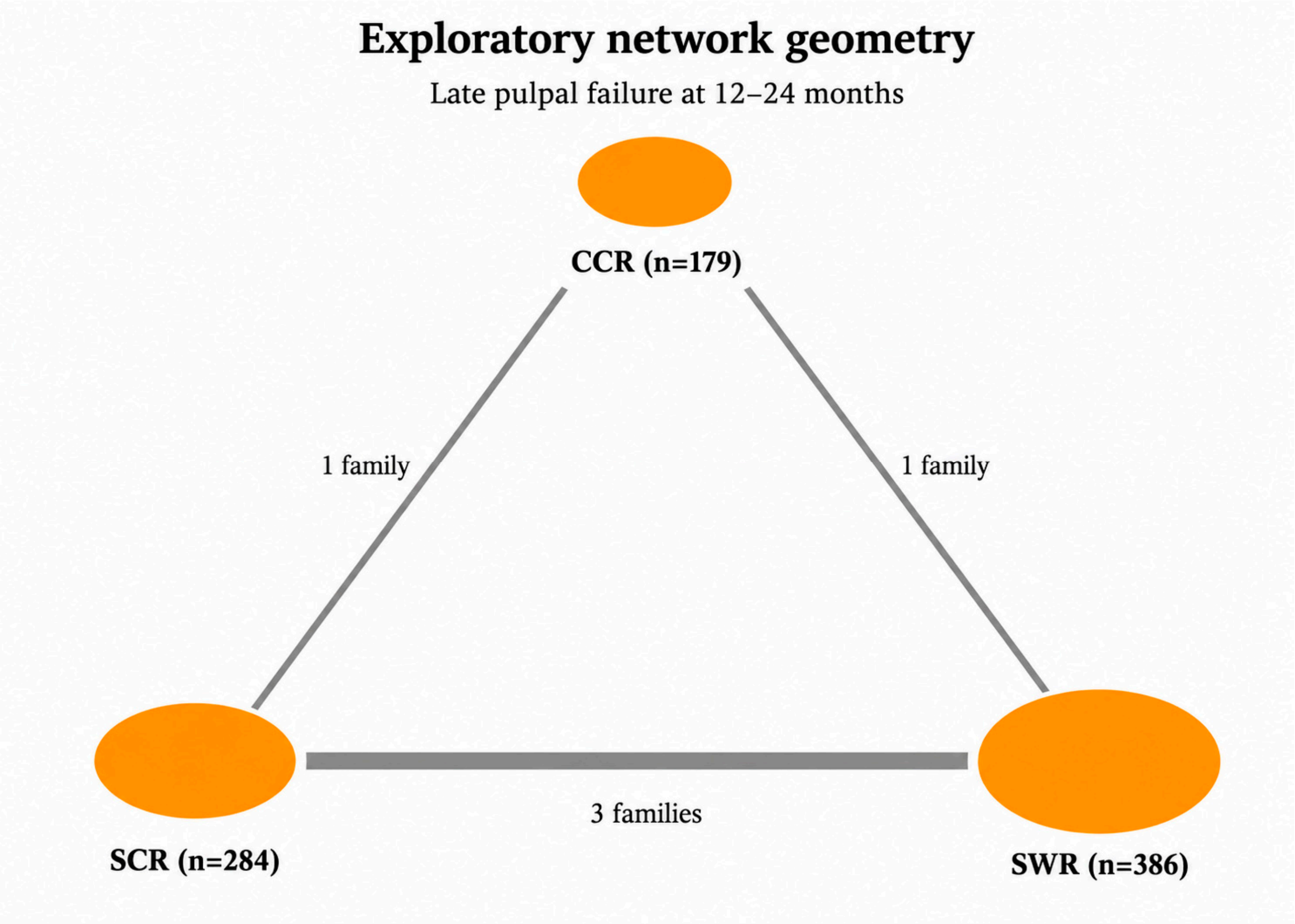

Figure S2. Outcome-specific network geometry for late pulpal failure at 12-24 months. Node size is proportional to the number of analyzed teeth in the RR-informative set; edge width and labels reflect independent trial families. Rando-Meirelles 2013 was double-zero and did not inform the RR network. The network was exploratory and was not used for ranking.

Table S11. Exploratory consistency, unrelated mean-effects, and inconsistency analyses for late pulpal failure at 12-24 months.

Analysis	Contrast / test	RR (95% CI) or statistic	p value	Additional information
Consistency model	SCR vs CCR	1.14 (0.12-10.85)	0.909	tau-squared = 1.30

Analysis	Contrast / test	RR (95% CI) or statistic	p value	Additional information
Consistency model	SWR vs CCR	1.73 (0.23-13.24)	0.597	tau-squared = 1.30
Consistency model	SCR vs SWR	0.66 (0.15-2.87)	0.578	tau-squared = 1.30
Unrelated mean-effects model	SCR vs CCR	5.00 (0.11-234.07)	0.412	tau-squared = 1.52
Unrelated mean-effects model	SWR vs CCR	0.96 (0.08-12.03)	0.976	tau-squared = 1.52
Unrelated mean-effects model	SCR vs SWR	0.50 (0.10-2.60)	0.409	tau-squared = 1.52
Inconsistency: Wald	Closed loop	chi-squared = 0.88; df = 1	0.347	Ratio of ratios = 0.096
Inconsistency: likelihood ratio	Consistency vs UME	chi-squared = 1.47; df = 1	0.226	Maximum-likelihood comparison

Note: Each of the five trial families contributed one effect, so the sampling-variance matrix was diagonal, and no within-family rho was required. Non-significant inconsistency tests do not establish consistency because the network had very low power.

Table S12. Differences between the registered protocol (PROSPERO) and the methods used in this review.

Topic	Registered/initial plan	Approach used in this review	Rationale	Timing/status
Registration chronology/status	Protocol submitted 4 March 2026 and registered 11 March 2026 (CRD420261321894).	The final database search was 20 January 2026. At submission, searching and screening were marked complete; extraction, RoB assessment, and synthesis were marked not started.	Distinguishes the registered stage declarations from the later completed review.	Registry dates and declared statuses are documented; registration was not prospective relative to searching/screening.
Outcome structure	Composite treatment-success outcome.	Intraoperative exposure, late pulpal failure, and restorative failure were analyzed separately; inseparable composites were descriptive only.	These endpoints represent distinct procedural, pulpal, and restorative constructs with different denominators and time windows.	Post hoc, after inspection of the preliminary composite analysis/effects and before the final checked final outcome-specific analysis and interpretation.
Primary evidence synthesis	Primary frequentist NMA with DerSimonian-Laird estimation and P-score ranking; pairwise analyses when data were sufficient.	Outcome-specific direct randomized evidence was primary. A supplementary REML network was restricted to late pulpal failure; no ranking or meta-regression was performed.	The network was sparse and clinically imbalanced; direct estimates were more auditable and did not support a treatment hierarchy.	Post hoc analytical refinement before the final checked final outcome-specific analysis and interpretation.
Direct pairwise pooling threshold	No explicit minimum number of independent trial families.	Pooling required at least three informative independent families; smaller contrasts remained study-specific.	Avoids unstable comparison-level pooling from one or two informative families.	Post hoc rule implemented before the final checked final outcome-specific analysis and interpretation.
Variance/interval estimation	DerSimonian-Laird estimation; no small-sample interval safeguard specified.	Eligible pooled models used REML with modified Knapp-Hartung inference.	Addresses small-sample uncertainty and prevents a Knapp-Hartung interval narrower than the corresponding Wald interval.	Post hoc method implemented in the final outcome-specific reanalysis.
Multiple reports/trial families	No explicit family-level rule specified.	One estimate per trial family, outcome, follow-up window, and contrast entered a synthesis.	Prevents double counting of repeated reports from one randomized cohort.	Implemented in the outcome-specific reanalysis; exact decision date was not separately recorded.
Participant-level clustering	No within-participant clustering method specified.	Adjusted estimates were preferred. Otherwise, design effects used a working ICC of 0.10 in the primary analysis and ICCs 0, 0.05, and 0.20 in sensitivity analyses.	Accounts for participants who could contribute more than one tooth while retaining the observed arm risks.	Implemented in the outcome-specific reanalysis; not prespecified.
Adjusted time-to-event estimates	No specific method stated for adjusted time-to-event effects.	Published adjusted HRs were retained in their reported metric, kept separate from binary RRs, and not pooled across outcomes/windows or repeated family reports.	Avoids mixing effect metrics, outcomes, time windows, and dependent follow-up reports.	Implemented in the outcome-specific reanalysis; not prespecified.

Topic	Registered/initial plan	Approach used in this review	Rationale	Timing/status
Effect-modifier analysis	Study-level exploration/meta-regression was contemplated within the network plan.	No meta-regression was performed; potential modifiers were compared qualitatively.	Seven families, with only one to three informative families per direct contrast, were inadequate for reliable meta-regression.	Removed before the final checked final outcome-specific analysis and interpretation.
Additional information sources	Published and unpublished studies would be sought.	No dedicated registry, gray literature, thesis, conference-proceeding, or other unpublished-study search was undertaken. Citation tracking supplemented database searching, but its date/yield were not separately preserved.	Reports the methods actually completed without presenting unverified search history as fact.	Searches completed 20 January 2026, before PROSPERO submission; disclosed as a deviation.
Population/root-maturity clarification	Permanent teeth in children, adolescents, or adults; root maturity was not explicit.	Reports explicitly confined to immature permanent teeth were excluded when root maturity was not reported by arm and no compatible subgroup was extractable. Chronological age alone did not exclude mixed-age permanent-tooth trials. Orhan included primary molars and young permanent first molars and provided no compatible extractable subgroup.	Avoids extrapolating an explicitly immature-only population to the clarified scope while retaining age-diverse permanent-tooth trials.	Post-registration scope clarification documented in the final eligibility audit; exact decision date was not recorded.

Note: At PROSPERO submission, searching and screening were complete, whereas extraction, risk-of-bias assessment, and synthesis were marked not started. The table separates documented dates/statuses from post hoc changes whose exact decision date was not preserved; items absent from the record are not presented as prespecified.

Table S13. GRADE evidence profile for the primary direct-evidence estimates.

Outcome	Contrast	Evidence base	Risk of bias	Inconsistency	Indirectness	Imprecision	Publication bias	Relative effect	Absolute effect per 1000	Certainty
Intraoperative pulp exposure	SCR vs SWR	583 teeth; 3 families	Serious (-1)	Not serious	Not serious	Very serious (-2)	Not downgraded	Pooled RR 0.11 (0.003-4.64)	42 with SWR; 5 with SCR (0-193); 37 fewer (42 fewer to 152 more)	Very low
Late pulpal failure, 12-24 months	SCR vs SWR	497 teeth; 3 families	Serious (-1)	Serious (-1)	Not serious	Very serious (-2)	Not downgraded	Pooled RR 0.50 (0.01-21.53)	91 with SWR; 45 with SCR (1-1000); 45 fewer (90 fewer to 909 more)	Very low
Intraoperative pulp exposure	SCR vs CCR	196 teeth; 2 families; not pooled	Serious (-1)	Not applicable	Not serious	Very serious (-2)	Not downgraded	Ahmed 0.07 (0.004-1.12); Khokhar 0.04 (0.002-0.63)	Not pooled	Very low
Intraoperative pulp exposure	SWR vs CCR	292 teeth; 1 family	Serious (-1)	Not applicable	Not serious	Serious (-1)	Not downgraded	RR 0.61 (0.39-0.94)	289 with CCR; 175 with SWR (113-270)	Low
Late pulpal failure, 12-24 months	SCR vs CCR	76 teeth; 2 families; 1 RR-informative	Serious (-1)	Not applicable	Not serious	Very serious (-2)	Not downgraded	Ahmed 5.00 (0.25-99.95); one double-zero	Not pooled	Very low
Late pulpal failure, 12-24 months	SWR vs CCR	292 teeth; 1 family	Serious (-1)	Not applicable	Not serious	Very serious (-2)	Not downgraded	RR 0.96 (0.45-2.04)	87 with CCR; 84 with SWR (40-178)	Very low
Restorative failure, 12-24 months	SCR vs SWR	480 teeth; 3 families; 1 RR-informative	Serious (-1)	Not applicable	Not serious	Very serious (-2)	Not downgraded	Recchi 1.75 (0.32-9.49); two double-zero	Not pooled	Very low

Note: Risk-of-bias judgments underpin the certainty assessments. Imprecision reflects the modified Knapp-Hartung intervals and the no-pooling rule; for late pulpal failure, the upper absolute-risk bound was capped at 1000 per 1000. The clinically unimportant range was RR 0.80-1.25.

Table S14. GRADE assessment of adjusted time-to-event estimates.

Report / outcome	Effect	Risk of bias	Inconsistency	Indirectness	Imprecision	Publication bias	Certainty	Rationale
Bjørndal 2017 [41] ; late pulpal failure, 60 months	HR 0.67 (0.45-1.05), SWR vs CCR	Serious (-1)	Not applicable	Not serious	Serious (-1)	Not downgraded	Low	One family; substantial missing long-term data; CI crosses no important difference
Maltz 2012 [45] ; late pulpal failure, 36 months	HR 0.21 (0.07-0.63), SCR vs SWR	Serious (-1)	Not applicable	Not serious	Not serious	Not downgraded	Moderate	Adjusted frailty model; one randomized family
Maltz 2018 [18] ; late pulpal failure, 60 months	HR 0.38 (0.23-0.63), SCR vs SWR	Serious (-1)	Not applicable	Not serious	Not serious	Not downgraded	Moderate	Adjusted frailty model; substantial attrition; same family as Maltz 2012
Jardim 2020 [46] ; restorative failure, 60 months	HR 0.83 (0.41-1.69), SCR vs SWR	Serious (-1)	Not applicable	Not serious	Very serious (-2)	Not downgraded	Very low	One family; substantial attrition; CI compatible with benefit and harm
Recchi 2024 [48] ; restorative failure, 18 months	HR 3.85 (0.58-25.00), SCR vs SWR	Serious (-1)	Not applicable	Not serious	Very serious (-2)	Not downgraded	Very low	Very wide CI; no explicit patient-level clustering adjustment

Note: Maltz 2012 []45 and Maltz 2018 [18] arose from the same randomized family and are not independent confirmations. Long-term estimates should be interpreted in light of substantial attrition. Composite HRs were recorded descriptively but were not graded because they did not correspond to a single outcome-specific endpoint.

Table S15. Manual CINeMA confidence assessment for the exploratory 12-24-month late pulpal-failure network.

Network comparison	NMA RR (95% CI); approximate prediction interval	Within-study bias	Across-studies bias	Indirectness	Imprecision	Heterogeneity	Incoherence	Overall confidence
SCR vs CCR	1.14 (0.12-10.85); 0.05-27.22	Major concerns	Some concerns	Major concerns	Major concerns	Major concerns	Major concerns	Very low
SWR vs CCR	1.73 (0.23-13.24); 0.08-35.33	Some concerns	Some concerns	Major concerns	Major concerns	Major concerns	Major concerns	Very low
SCR vs SWR	0.66 (0.15-2.87); 0.05-9.61	Some concerns	Some concerns	Major concerns	Major concerns	Major concerns	Major concerns	Very low

Note: CINeMA judgments used the RR 0.80-1.25 equivalence range. Major concerns reflected marked imbalance in potential effect modifiers, very wide confidence and prediction intervals, substantial common heterogeneity, and directionally discordant direct and indirect estimates. Low-power non-significant tests did not establish coherence.
